# Electrochemical Hydroformylation with a Homogeneous Rhodium Catalyst

**DOI:** 10.1021/jacs.6c15582

**Published:** 2026-10-01

**Authors:** Emma L. Cosner, Spencer P. Delgado-Kukuczka, Haochen Zhang, Abigail L. Blenko, Evan V. Miu, Karthish Manthiram

**Affiliations:** Division of Chemistry and Chemical Engineering, 6469 California Institute of Technology, Pasadena, California 91125, United States

## Abstract

Electrochemical hydroformylation is an attractive pathway to synthesize aldehydes at ambient conditions using electrons sourced from renewable energy as a reductant instead of hydrogen gas sourced from energy-intensive steam methane reforming. However, previously reported electrochemical hydroformylation systems have shown limited rates and selectivities using heterogeneous catalysts, motivating continued development. In this work, we report a homogeneously catalyzed system that achieved a maximum partial current density of 1.1 ± 0.1 mA cm^–2^ and a Faradaic efficiency of 29 ± 1% toward the conversion of styrene to 2-phenylpropanal at 5 bar CO and 25 °C. Infrared and UV-visible spectroscopies suggested that the initial Rh_2_Cl_2_(CO)_4_ precatalyst undergoes pressure-dependent speciation to Rh­(I)–CO species in the presence of CO. Kinetic experiments revealed a complex mechanism in which the rate-determining step likely shifts between electron transfer, substrate coordination, and Rh–H bond cleavage in different substrate concentration and overpotential regimes. Looking forward, the insight from this work informs future strategies to improve the rate and selectivity of electrochemical hydroformylation and other electrochemical C–C bond formation reactions.

## Graphical abstract

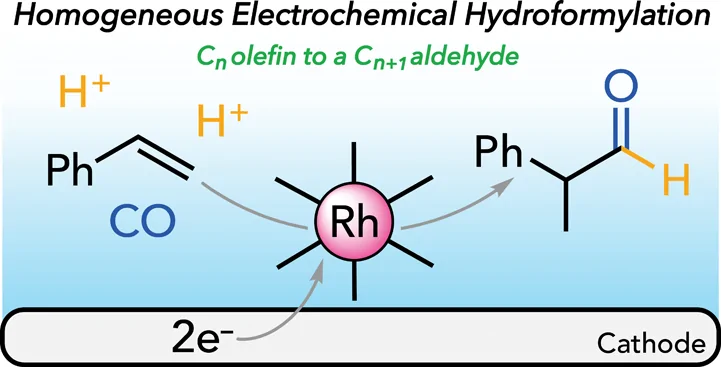

## 1 Introduction

Thermochemical hydroformylation (thermo-HFN) is a C–C bond formation reaction in which C_n_ olefin substrates are coupled with carbon monoxide (CO) and hydrogen (H_2_), a mixture known as syngas, to form C_n+1_ aldehydes. Annually, this process produces over 10 million tons of aldehydes, which are utilized as precursors for the synthesis of plasticizers, surfactants, and solvents.^1^ Industrial thermo-HFN is driven by elevated temperatures (90–120 °C) and pressures (10–60 bar), the latter of which necessitates large-scale, centralized operation (Figure 1A).^2^ The H_2_ required for thermo-HFN is sourced from steam methane reforming, an energy-intensive process that requires high temperatures (700–1000 °C) generated by burning fossil fuels.^3^ Electrifying hydroformylation allows abundant, low-cost inputs such as H_2_O and renewable electricity to be used as the hydrogen source in the form of protons and electrons instead of H_2_ (Figure 1B). In electrochemical hydroformylation (electro-HFN), applied voltage acts as a powerful driving force that can unlock mild operating conditions.

**Figure 1:**
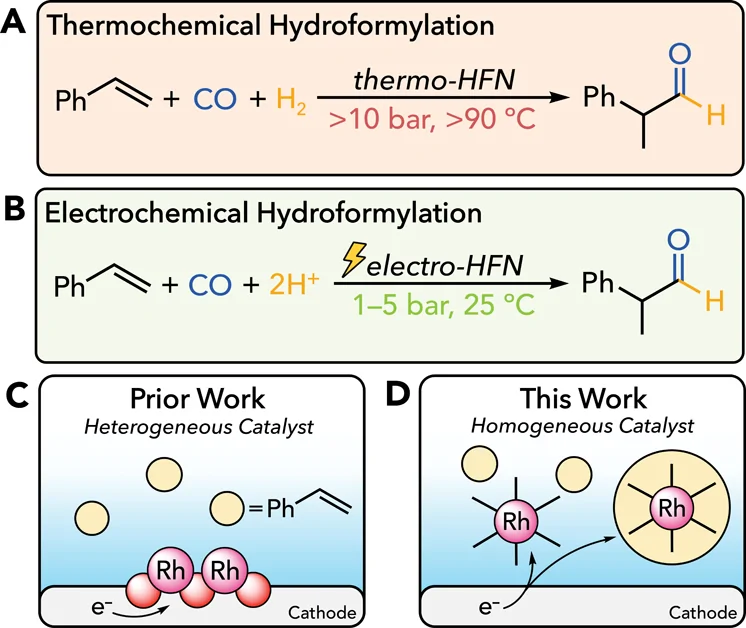
Electrification enables the use of near-ambient pressures and temperatures and avoids hydrogen gas, sourced from steam methane reforming, as a feedstock. **A**) Schematic of thermochemical hydroformylation (thermo-HFN) at standard operating conditions. **B**) Schematic of electrochemical hydroformylation (electro-HFN) at standard operating conditions. **C**) Prior reports using heterogeneous Rh-based oxide materials for electro-HFN of styrene in a phase-separated electrolyte. **D**) This work using homogeneous rhodium complexes as precatalysts for styrene electro-HFN in a phase-separated electrolyte.

The first report of electro-HFN demonstrated that heterogeneous noble metal chloride catalysts can facilitate the conversion of ethylene to propionaldehyde.^4^ However, this reaction required elevated temperatures (80–120 °C) and still utilized H_2_ as a reactant for hydrogen oxidation at the counter electrode. This system achieved a maximum current density of 0.1 mA cm^–2^ and a Faradaic efficiency (FE) of 5% for the hydroformylation of ethylene. Our group recently demonstrated that heterogeneous rhodium on cerium oxide (Rh@CeO_2_) and rhodium oxide (Rh_2_O_3_) electrocatalysts can facilitate the conversion of styrene to 2-phenylpropanal at ambient conditions without added H_2_ (Figure 1C).^5^ This system achieved a maximum partial current density of 0.05 mA cm^–2^ and FE of 15% using Rh@CeO_2_ in a phase-separated electrolyte. Despite these pioneering efforts, reaction rates are not yet competitive with the current industrial thermo-HFN process. Notably, in the Rh@CeO_2_ system, addition of a strong acid such as trifluoromethanesulfonic acid (HOTf) was necessary to observe any reactivity, which presumably contributed to the low FEs (≤15%) due to increased parasitic hydrogen evolution reaction (HER). In addition, the details of the mechanism remained unclear, preventing further enhancement of electro-HFN systems.

Zeng et al. found that Rh@CeO_2_ resembles a Rh­(III) species under reaction conditions.^5^ However, the typical active species reported in homogeneous and heterogeneous thermo-HFN literature are Rh­(I) and low-valent Rh, respectively.^6–11^ Furthermore, Rh centers in Rh@CeO_2_ were found to be bound to 4.5 to 5 lattice oxygens under applied potential. This coordinative saturation of Rh centers in Rh@CeO_2_ could have contributed to low rates because the migratory insertion steps in the hydroformylation mechanism require that the Rh center bind two substrates at once. We hypothesized that homogeneous Rh complexes could achieve higher partial current densities than heterogeneous catalysts due to their undersaturated, tunable coordination environment and the accessibility of low-valent Rh centers.

Herein, we report a homogeneously catalyzed electro-HFN system that achieved a partial current density and FE of 1.1 ± 0.1 mA cm^–2^ and 29 ± 1%, respectively (Figure 1D). We used homogeneous Rh complexes as precatalysts that partitioned into the organic or aqueous phase of a biphasic electrolyte. Interaction of these solution species with the cathode results in Rh reduction and the formation of Rh–H species. Then, the electrochemically generated Rh–H species undergo subsequent thermochemical reactions with styrene and CO to generate the desired aldehyde product. Notably, this system can operate without addition of a strong acid, which mitigates parasitic HER. Through reaction condition screening, we found that the combination of di-μ-chloro-tetracarbonyldirhodium­(I) (Rh_2_Cl_2_(CO)_4_) as the precatalyst, hydrophobic carbon paper (CP) as the cathode, tetrabutylammonium trifluoromethanesulfonate (TBAOTf) as the supporting electrolyte salt, and isopropanol (IPA) and H_2_O as cosolvents resulted in the highest partial current density compared to other conditions. Additionally, we found that Rh_2_Cl_2_(CO)_4_ underwent a chemical reaction in the presence of CO, as reflected by a change in the color of the electrolyte solution. Infrared and UV-visible spectroscopies were used to track the speciation of Rh_2_Cl_2_(CO)_4_, suggesting its conversion to Rh­(I)–CO species in the presence of CO. Finally, we conducted kinetic analysis to determine the interdependence between reactant concentrations and applied potential in electro-HFN. We found that the rate-determining step changes across various substrate concentration and overpotential regimes.

## 2 Results and Discussion

### 2.1 Low-Valent Rhodium, Biphasic Electrolytes, Hydrophobic Interfaces, and Organic Cosolvents Enable High Rates

We explored various Rh complexes and reaction conditions to understand what characteristics govern the rate and selectivity of homogeneous electro-HFN. In homogeneous and heterogeneous thermo-HFN, organic solvents are used to improve the solubility of nonpolar substrates and can heavily influence substrate adsorption and binding, as well as intermediate stabilization.^12,13^ Electrochemical reactions instead require an ionically conductive reaction medium, which is typically polar. Electrochemical reactions also introduce additional variables beyond solvent, such as the electrode material and supporting electrolyte salt, which both affect the reaction environment. We hypothesized that these additional variables could reconcile the opposing reaction medium requirements. Specifically, the hydrophobicity of the reaction environment could be tuned through the choice of cathode material, organic cosolvent, and supporting electrolyte salt, all while maintaining an ionically conductive electrolyte.^14–16^

Styrene is a canonical model substrate in thermo-HFN literature and was used in a blended organic solvent and H_2_O electrolyte.^11,17–23^ Notably, we used a styrene concentration of 0.52 M, which is beyond the solubility limit of styrene in the blended organic solvent and H_2_O solution. The resulting electrolyte was phase-separated with an organic styrene phase and an aqueous phase. The phase-separated electrolyte was stirred with a magnetic stir bar at 800 rpm during electrolysis. Chronoamperometry experiments were conducted in a custom PEEK sandwich cell at static 5 bar CO pressure and an *iR*-corrected applied potential of −0.91 V versus the standard hydrogen electrode (SHE) (Figure S1). All potentials are referenced against SHE unless stated otherwise. A titanium felt coated with ruthenium iridium oxide was used as the counter electrode and an Ag/AgCl leakless electrode was used as the reference electrode. Each experiment was conducted with 0.1 M of the supporting electrolyte salt and 3 mM of the homogeneous Rh complex. Reaction products were quantified by gas chromatography mass spectrometry (GC-MS) and ^1^H nuclear magnetic resonance (NMR) spectroscopy using 1,3,5-trimethoxybenzene and formic acid as internal standards, respectively (Figures S2 and S5). The production rate of the major product, 2-phenylpropanal, was used to calculate the partial current densities and FEs toward electro-HFN in the following discussion (eqs S1 and S2).

Based on the precedent of thermo-HFN, we hypothesized that Rh­(I) complexes would possess the highest activity for electro-HFN (vide supra). This hypothesis was tested by evaluating commercially available Rh complexes with diverse oxidation states and coordination environments for their ability to act as precatalysts for electro-HFN (Figure 2A). Consistent with our hypothesis, we found that Rh­(I) complexes produced higher rates and FEs compared to Rh­(III) and Rh­(II) complexes. When partial current densities were normalized on a per Rh atom basis, dinuclear Rh­(I) complexes still outperformed Rh­(III) and Rh­(II) complexes (Figure S8).

**Figure 2:**
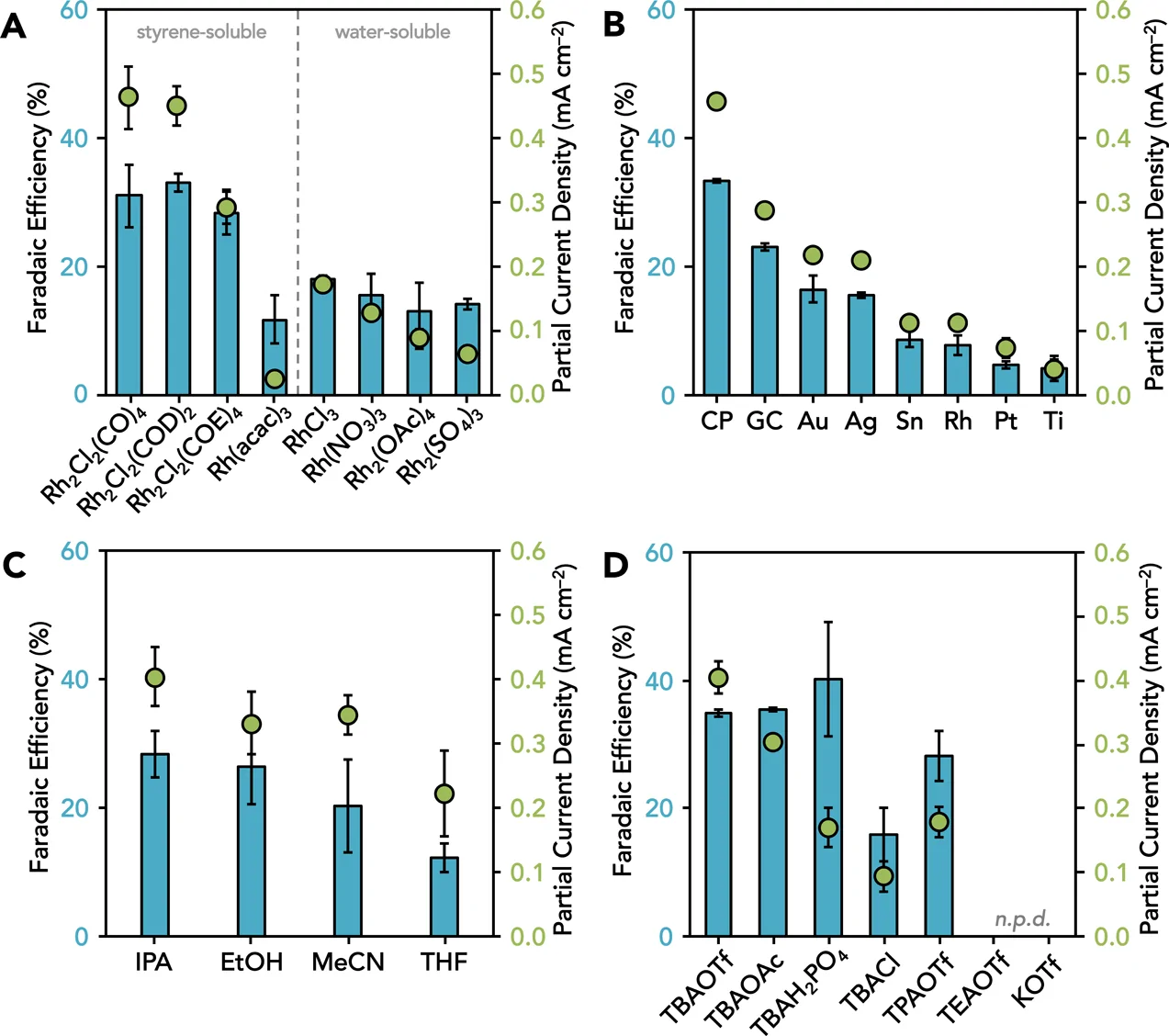
Partial current density and Faradaic efficiency for reaction condition screening. Error bars represent one standard deviation with n ≥ 2. **A**) Rh complexes and their styrene/water solubility; di-μ-chloro-tetracarbonyldirhodium­(I) (Rh_2_Cl_2_(CO)_4_), chloro­(1,5-cyclooctadiene)­rhodium­(I) dimer (Rh_2_Cl_2_(COD)_2_), chlorobis­(cyclooctene)­rhodium­(I) dimer (Rh_2_Cl_2_(COE)_4_), rhodium­(III) acetylacetonate (Rh­(acac)_3_), rhodium­(III) chloride hydrate (RhCl_3_·xH_2_O), rhodium­(III) nitrate hydrate (Rh­(NO_3_)_3_·xH_2_O), rhodium­(II) acetate dimer (Rh_2_(OAc)_4_), and rhodium­(III) sulfate (Rh_2_(SO_4_)_3_). **B**) Cathodes: Toray 120 carbon paper (CP), glassy carbon (GC), gold (Au), silver (Ag), tin (Sn), rhodium (Rh), platinum (Pt), and titanium (Ti) foils. **C**) Solvents: isopropanol (IPA), ethanol (EtOH), acetonitrile (MeCN), and tetrahydrofuran (THF). **D**) Supporting electrolyte salts: tetrabutylammonium trifluoromethanesulfonate (TBAOTf), tetrabutylammonium acetate (TBAOAc), tetrabutylammonium dihydrogen phosphate (TBAH_2_PO_4_), tetrabutylammonium chloride (TBACl), tetrapropylammonium trifluoromethanesulfonate (TPAOTf), tetraethylammonium trifluoromethanesulfonate (TEAOTf), and potassium trifluoromethanesulfonate (KOTf); n.p.d. = no product detected. Experimental conditions unless stated otherwise: 3 mM Rh_2_Cl_2_(CO)_4_, 0.52 M styrene, 0.1 M TBAOTf, 50/50 v/v IPA/H_2_O, Toray 120 (5% Teflon) carbon paper cathode, 5 bar CO, −0.91 V vs. SHE.

When the styrene concentration was low enough to form a single-phase aqueous electrolyte (0.28 M), we observed deposits on the cathode after electrolysis at 5 bar CO. However, when the styrene concentration was increased to form a phase-separated electrolyte (0.52 M), several of the tested complexes partitioned into the styrene phase instead of the aqueous phase. These complexes were resistant to deposit formation at CO pressures as low as 2 bar. By contrast, complexes that remained in the aqueous phase at 0.52 M styrene still formed deposits at 5 bar CO (Figure S9 and Supplemental Discussion 1). Scanning electron microscopy coupled with energy-dispersive X-ray spectroscopy (SEM-EDS) confirmed that deposits from both cases contained Rh (Figures S10 and S11). X-ray photoelectron spectroscopy (XPS) depth profiling on these cathodes revealed the presence of Rh­(III) and Rh(0) components, consistent with Rh_2_O_3_ and Rh metal (Figure S12). Since the electrolysis was conducted at a large overpotential with respect to Rh plating (*E*°(Rh^3+/0^) = 0.76 V and *E*°(Rh^+/0^) = 0.60 V),^24^ these deposits likely originated from reduction of the homogeneous Rh species to Rh(0), which underwent surface oxidation in air during XPS sample preparation. To exclude the possibility of these Rh deposits being catalytically active, we ran an electrolysis using a cathode with Rh deposits in electrolyte containing no additional Rh precatalyst. No 2-phenylpropanal was generated (Figure S13), suggesting that the Rh deposits on the cathode are not catalytically active for electro-HFN. For subsequent experiments, a biphasic electrolyte was employed as an effective strategy to prevent electrodeposition of homogeneous species.

Among the complexes evaluated, Rh_2_Cl_2_(CO)_4_ produced the highest partial current density toward electro-HFN and was therefore used as the precatalyst for the rest of the condition screening. To probe the relationship between the concentration of the precatalyst and the partial current density, we tested Rh_2_Cl_2_(CO)_4_ concentrations from 0.75 to 7 mM. We observed a monotonic increase in the partial current density with precatalyst concentration (Figure S14). From this plot, we extracted a Rh_2_Cl_2_(CO)_4_ rate order of 0.82 ± 0.03. Deviation from unity can be explained by concentration-dependent speciation and conversion of the precatalyst to a form that is not catalytically active (Section 2.4). Notably, at 7 mM, we achieved the highest partial current density in this study, 1.1 ± 0.1 mA cm^–2^, with an FE of 29 ± 1%.

Then, we tested the effect of cathode material on the reaction rate and selectivity. We found that carbonaceous cathodes, such as CP and glassy carbon (GC), yielded the highest partial current densities and FEs compared to metal foil cathodes (Figure 2B). This observation could be explained by the increased interaction of carbonaceous cathodes with the styrene phase due to their hydrophobic surfaces.^25–27^ The lower partial current density and FE observed with metal foils could be due to their relatively lower hydrophobicity, ability to facilitate HER, or decreased site availability due to CO poisoning.^28,29^ CP was chosen as the cathode for subsequent experiments because it produced the highest partial current density, which could be attributed to its high surface area (Figure S15 and Supplemental Discussion 2).

Next, we screened acetonitrile (MeCN), tetrahydrofuran (THF), ethanol (EtOH), and IPA as cosolvents in a 50/50 volume/volume mixture with H_2_O (Figure 2C). We found that IPA produced higher partial current densities than EtOH, MeCN, and THF. While solvent identity can have complex effects on solvation structures, reactant solubility, and transition state energies, we suspect that this trend can be mostly attributed to the cosolvents’ ability to blend the organic and aqueous phases of the electrolyte, thus facilitating the distribution of chemical species between the two phases. All phase-separated cosolvent electrolytes were stirred during electrolysis. However, once stirring was stopped in the MeCN and THF systems, the styrene phase immediately coalesced and separated from the aqueous phase, indicating that these cosolvents could not effectively mix the organic and aqueous phases. On the other hand, electrolytes containing EtOH and IPA cosolvents remained mixed even after the stirring was stopped, evident from the cloudiness of the mixture. Between EtOH and IPA, higher partial current densities and FEs in IPA are likely due to its greater hydrophobicity, which facilitated better mixing between the organic and aqueous phases. IPA produced the highest partial current density and therefore was used as the organic cosolvent for subsequent experiments.

Finally, we investigated the influence of the supporting electrolyte salt and found that the identity of the cation strongly impacted reactivity (Figure 2D). When using trifluoromethanesulfonate (OTf^–^) as the anion, tetrabutylammonium (TBA^+^) achieved a higher partial current density than tetrapropylammonium (TPA^+^), while tetraethylammonium (TEA^+^) did not exhibit any detectable reactivity. When an inorganic cation such as K^+^ was used, no product was detected. We suspect that longer carbon chains enhance reactivity by increasing the hydrophobicity of the double layer, emulsifying the styrene and aqueous layers, or acting as a phase-transfer agent for the Rh catalyst.^30–37^ All three explanations are consistent with the partial current density dependence on TBA^+^ concentration, which rises and saturates near 0.1 M at constant ionic strength (Figure S16). When screening anions in TBA^+^ salts, we found that the anion influenced electro-HFN performance in two ways: through the speciation of the Rh precatalyst and through the phase behavior of the electrolyte. UV-visible (UV-Vis) data indicated that the Rh precatalyst underwent a transformation under CO in the presence of OTf^–^, OAc^–^, or H_2_PO_4_^–^, but not with Cl^–^ (Section 2.4). We attribute the low partial current density and FE of electrolytes containing TBACl to this difference. Among the three anions that facilitated this transformation, only OTf^–^ maintained a phase-separated electrolyte at 0.52 M styrene, while OAc^–^ and H_2_PO_4_^–^ produced a single phase. These two properties likely account for the higher partial current density at comparable FE obtained with TBAOTf, which was used for subsequent experiments.

The combination of Rh_2_Cl_2_(CO)_4_ as the precatalyst, TBAOTf as the supporting electrolyte salt, IPA as the cosolvent, and CP as the cathode achieved a partial current density and FE of 0.46 ± 0.05 mA cm^–2^ and 31 ± 5%, respectively, at −0.91 V vs. SHE and was used for subsequent kinetic analysis.

### 2.2 Rh_2_Cl_2_(CO)_4_ Is More Selective for 2-Phenylpropanal than H_2_ or Ethylbenzene

The major product detected was the branched aldehyde, 2-phenylpropanal, while the linear aldehyde, 3-phenylpropanal, was observed in trace amounts. The observed regioselectivity is consistent with previous studies of electro-HFN with styrene.^5^ The onset potential for product formation was −0.66 V, which is consistent with the onset potential observed in cyclic voltammetry (Figure S17). We also detected side products including ethylbenzene from styrene hydrogenation and H_2_ from HER. At −1.56 V vs. SHE, a trace amount of the hydrogenated aldehyde product, 2-phenyl-1-propanol, was also observed. H_2_ was quantified via online gas chromatography with a thermal conductivity detector (GC-TCD) (Figure S6). The partial current densities of both 2-phenylpropanal and ethylbenzene increased as the potential decreased from −0.66 V to −1.11 V and remained relatively constant from −1.11 V to −1.56 V (Figure 3A). By contrast, the rate of HER increased monotonically with increasing overpotential. The FE for 2-phenylpropanal peaked at 42 ± 6% at −0.96 V and gradually decreased with increasing overpotential (Figure 3B). Across most of the potential range studied (−0.66 V to −1.46 V), Rh_2_Cl_2_(CO)_4_ was more selective for 2-phenylpropanal than ethylbenzene and H_2_ combined.

**Figure 3:**
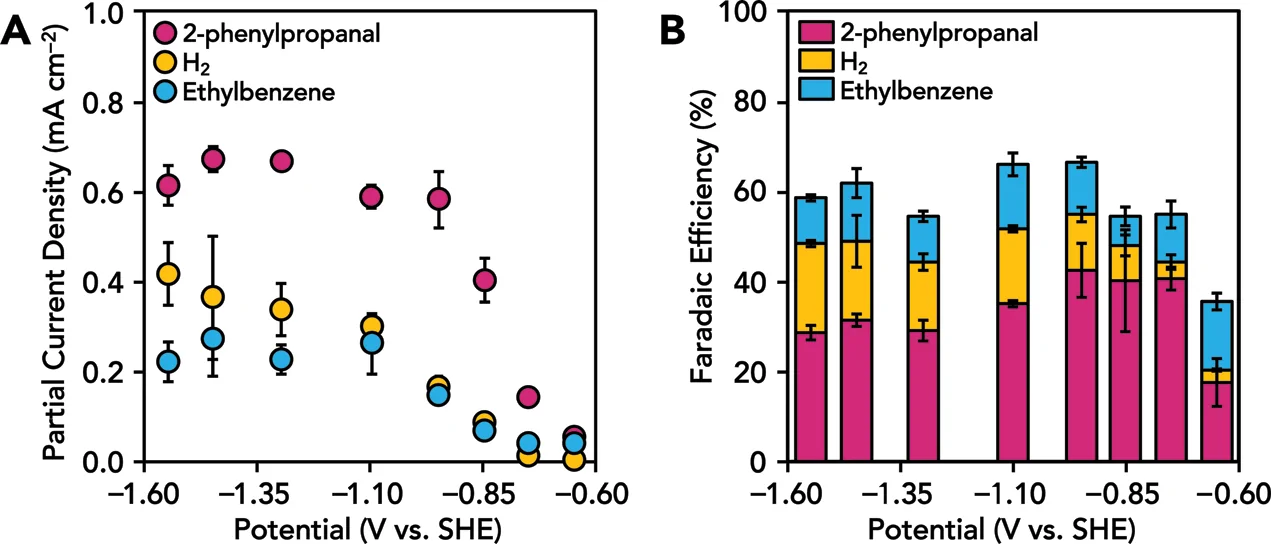
Potential-dependent selectivity of electro-HFN toward 2-phenylpropanal, H_2_, and ethylbenzene. Error bars represent one standard deviation with n ≥ 2. **A**) Partial current density as a function of applied potential. **B**) Faradaic efficiency as a function of applied potential. Experimental conditions unless stated otherwise: 3 mM Rh_2_Cl_2_(CO)_4_, 0.52 M styrene, 0.1 M TBAOTf, 50/50 v/v IPA/H_2_O, Toray 120 (5% Teflon) carbon paper cathode, 5 bar CO.

### 2.3 Control Experiments Reveal a Dominant Electrochemical Mechanism for Aldehyde Generation

Due to the presence of H_2_ as a side product, it was possible that 2-phenylpropanal was generated through a thermochemical pathway with electrochemically generated H_2_. In this case, potential dependence could arise due to the modulation of H_2_ concentration in the electrolyte. To exclude this possibility, we performed a series of control experiments with externally supplied H_2_. To compare the reaction rates of electrochemical and thermochemical experiments, turnover frequency (TOF) was used to represent the rate of product formation instead of partial current density. At 2.5 bar CO and 2.5 bar H_2_ without applied potential (open circuit potential, OCP), 2-phenylpropanal was produced at a TOF of 0.017 ± 0.004 h^–1^, only 2.0% of that obtained under 2.5 bar CO with applied potential (0.86 ± 0.12 h^–1^) (Figure 4A, left and middle). Notably, the equilibrium H_2_ concentration under 2.5 bar CO and 2.5 bar H_2_ was comparable to the maximum interfacial concentration of in situ generated H_2_ under 2.5 bar CO with applied potential, as estimated from a transport-based calculation assuming all charge goes to HER (Supplemental Discussion 3). Therefore, the difference in production rates between these conditions likely arises from the presence of an applied potential rather than differences in H_2_ concentrations.

**Figure 4:**
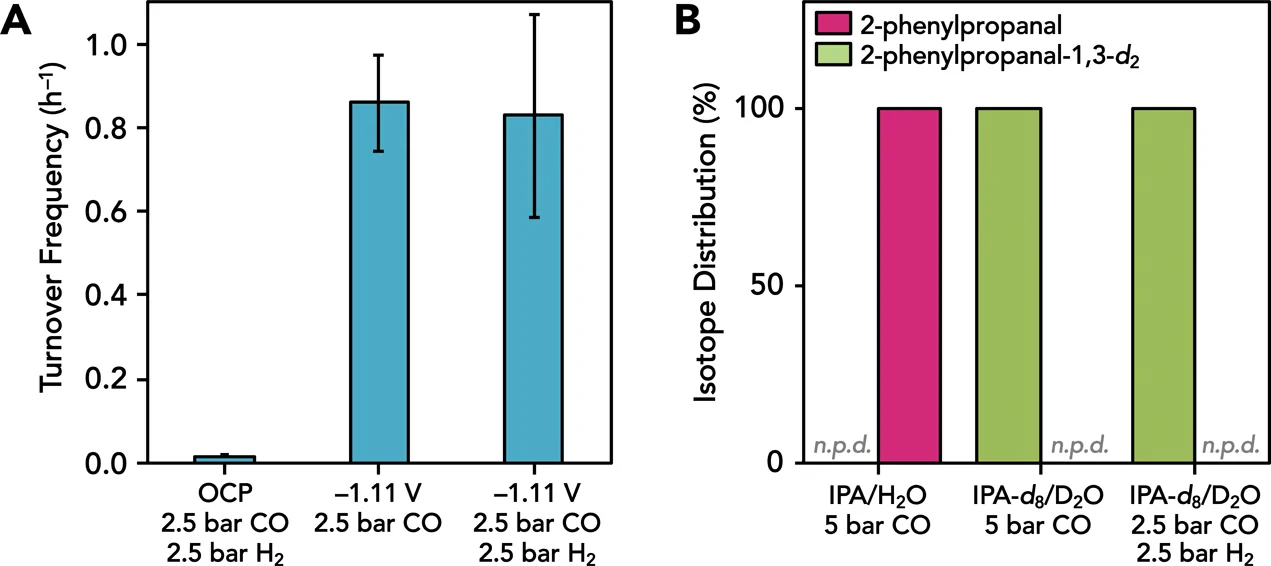
Thermochemical and electrochemical controls. Error bars represent one standard deviation with n ≥ 2. **A**) Turnover frequency of the catalyst during a thermochemical experiment (left), an electrochemical experiment without external H_2_ (middle), and an electrochemical experiment with external H_2_ (right). **B**) Isotope distribution of 2-phenylpropanal in the presence of protonated solvent (left), deuterated solvent (middle), and deuterated solvent and H_2_ gas (right); n.p.d. = no product detected. Experimental conditions unless stated otherwise: 3 mM Rh_2_Cl_2_(CO)_4_, 0.52 M styrene, 0.1 M TBAOTf, 50/50 v/v IPA/H_2_O, Toray 120 (5% Teflon) carbon paper cathode, 2.5 bar CO, −1.11 V vs. SHE.

Additionally, we conducted electrolysis at 2.5 bar CO with and without additional 2.5 bar H_2_ (Figure 4A, middle and right). If the thermochemical mechanism were the dominant pathway for generating 2-phenylpropanal, we would expect that adding external H_2_ to an electrochemical experiment would increase the observed production rate. However, we observed no significant difference in production rates upon addition of H_2_. In principle, this behavior could arise from a thermochemical mechanism with an apparent zero-order dependence on H_2_. However, we consider this to be unlikely because this mechanism would not account for the dependence of 2-phenylpropanal production on applied potential.

Moreover, when conducting experiments in deuterated solvent (50/50 v/v IPA-*d*_8_/D_2_O) at 2.5 bar CO with and without 2.5 bar H_2_, we observed only the deuterated product by GC-MS (Figure 4B). This suggests that H_2_ is not directly involved in the reaction. Instead, the protons incorporated into 2-phenylpropanal originate from the solvent through electrolysis.

Further support for an electrochemical mechanism arises from the reaction rate dependence on styrene concentration (Section 2.5). We observed that the total current density doubled when the styrene concentration was increased from 0.016 to 0.13 M (Figure S18). This trend is inconsistent with a purely thermochemical mechanism in which only HER accounts for the total current. HER should be either unaffected by styrene or inhibited by competitive binding, so the total current should not increase with styrene concentration. The increase we observed therefore indicates that styrene is directly involved in the electrochemical reaction. Taken together, these data provide evidence that an electrochemical mechanism is the dominant pathway for 2-phenylpropanal generation in this homogeneous system, rather than a thermochemical mechanism via electrochemically generated H_2_.

### 2.4 Rh_2_Cl_2_(CO)_4_ Undergoes a Transformation in the Presence of CO

Upon pressurization of the electrolyte with CO before electrolysis, we observed that Rh_2_Cl_2_(CO)_4_ underwent distinct color changes and a precipitate was formed (Figure S19). Initially, Rh_2_Cl_2_(CO)_4_ dissolved in styrene was orange and became a yellow cloudy mixture upon addition of 0.1 M TBAOTf in 50/50 v/v IPA/H_2_O. Upon bubbling 1 bar CO through the electrolyte, the mixture turned purple. With further pressurization above 1 bar, the color progressively shifted to purple-red and became darker as the pressure increased to 5 bar.

The precipitate was collected via centrifugation and dried at room temperature. Characterization of the powder using X-ray diffraction (XRD) revealed that this species corresponded to hexarhodium hexadecacarbonyl (Rh_6_(CO)_16_) (Figure 5A). It has been previously reported that Rh_2_Cl_2_(CO)_4_ can undergo reduction by CO to form Rh(0) carbonyl complexes in the presence of H_2_O, producing stoichiometric amounts of HCl and CO_2_.^38,39^ Given that Rh_6_(CO)_16_ is a known thermo-HFN catalyst, we performed a control experiment to determine if this species could also act as an electro-HFN catalyst.^40–47^ The precipitate was collected after the electrolyte was pressurized at 5 bar CO for 30 min, which represented the maximum amount of Rh_6_(CO)_16_ that could be formed during a typical electrolysis. Then, the precipitate was suspended in electrolyte containing no Rh_2_Cl_2_(CO)_4_ and an electrolysis was performed at −1.11 V vs. SHE. This condition produced a partial current density of 0.021 ± 0.004 mA cm^–2^, which was only 4% of the partial current density produced when the electrolyte contained the Rh_2_Cl_2_(CO)_4_ precatalyst (Figure 5B). These data suggest that Rh_6_(CO)_16_ generated under reaction conditions is only a minor contributor to 2-phenylpropanal formation and that the Rh species in the styrene phase is the major catalytically relevant species. For subsequent characterization, the precipitate was removed by centrifugation to study the remaining species in the styrene phase.

**Figure 5:**
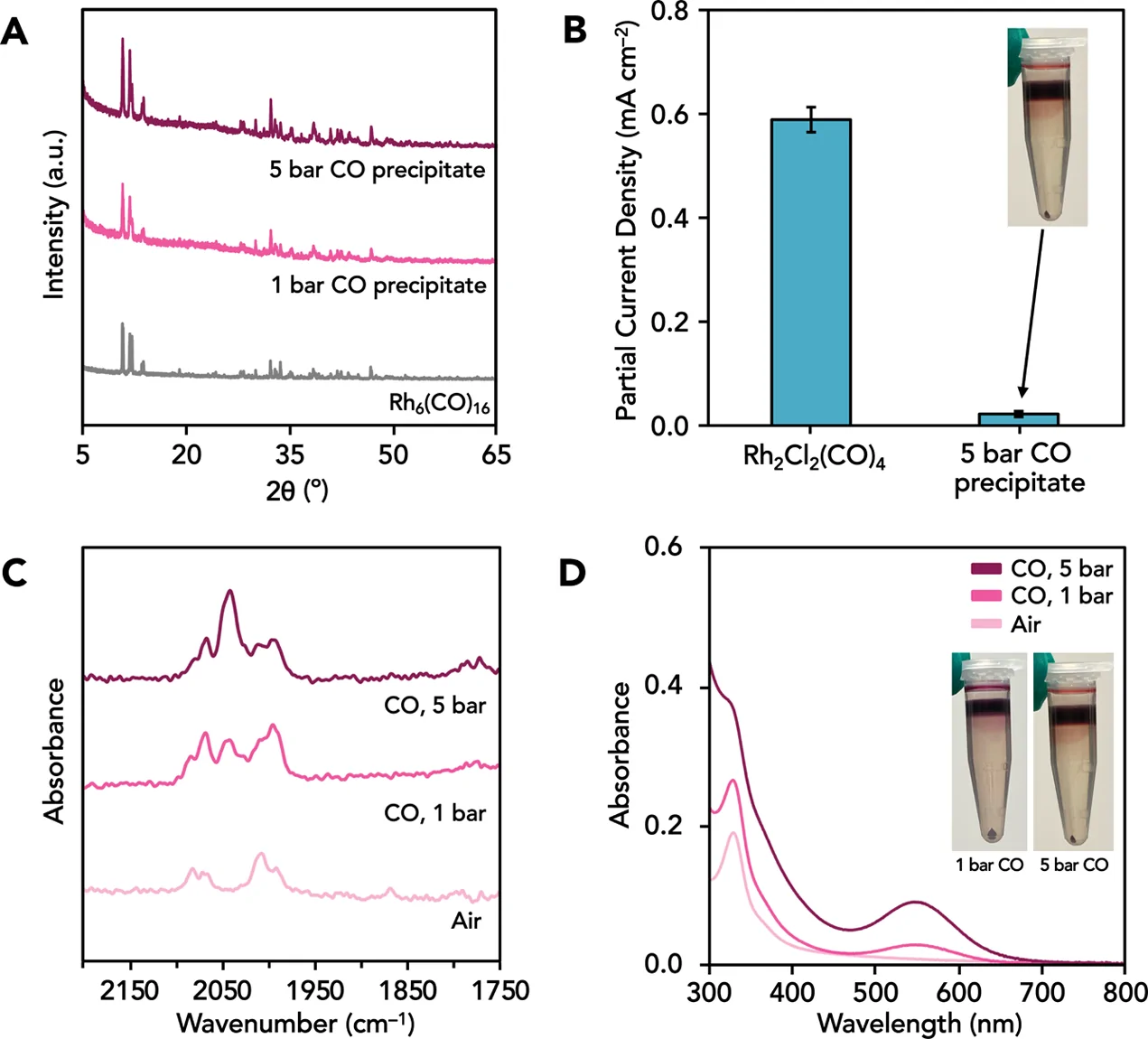
Characterization of the precipitate (**A–B**) and Rh species present in the styrene phase (**C–D**) evolved from the Rh_2_Cl_2_(CO)_4_ precatalyst in the presence of CO. These species were separated via centrifugation of the electrolyte at 19,090 rcf for 5 min. **A**) X-ray diffraction pattern of the precipitate collected at 1 and 5 bar CO and their comparison to a measured pattern of commercial Rh_6_(CO)_16_. **B**) Control experiment using the Rh_6_(CO)_16_ precipitate in an electrolysis. Error bars represent one standard deviation with n ≥ 2. **C**) Attenuated total reflectance Fourier transform infrared absorption spectroscopy of the styrene phase after pressurization at 1 and 5 bar CO. **D**) UV-visible spectroscopy of the styrene phase diluted in IPA after pressurization at 1 and 5 bar CO. Experimental conditions unless stated otherwise: 3 mM Rh_2_Cl_2_(CO)_4_, 0.52 M styrene, 0.1 M TBAOTf, 50/50 v/v IPA/H_2_O, Toray 120 (5% Teflon) carbon paper cathode, 5 bar CO.

We characterized the Rh species in the styrene phase using ex situ attenuated total reflectance Fourier transform infrared (ATR-FTIR) absorption spectroscopy. Without external CO, Rh_2_Cl_2_(CO)_4_ exhibited four main features in the terminal carbonyl region (*ν*_CO_ = 2083, 2068, 2009, 1992 cm^–1^), which are consistent with the asymmetric and symmetric stretches of Rh­(CO)_2_ species (Figure 5C).^48–52^ Upon bubbling 1 bar CO through the electrolyte, these features remained and a new peak emerged at 2044 cm^–1^, which intensified when the CO pressure was increased to 5 bar. The feature is most consistent with a terminal CO bound to an electron-rich Rh center.^53^ This spectral progression suggests that there was a mixture of Rh species present whose ratio was dictated by CO pressure. The wavenumbers of these terminal carbonyl stretches were inconsistent with commonly reported monomeric structures such as [Rh­(CO)_4_]^+^, [RhCl_2_(CO)_2_]^−^, or RhCl­(CO)_3_. These species were therefore excluded from consideration.^48,54–56^ Furthermore, the absence of any significant peaks corresponding to bridging carbonyls between 1900 and 1780 cm^–1^ excluded the possibility that the solution contained Rh_4_(CO)_12_, Rh_6_(CO)_16_, Rh(0) nanoclusters, or related anionic complexes.^52,57–61^

Then, the color changes of the Rh species were characterized by ex situ UV-Vis spectroscopy. In the absence of external CO, Rh_2_Cl_2_(CO)_4_ was orange and possessed an absorption peak around 330 nm, which is consistent with a metal-to-ligand charge transfer (MLCT) characteristic of a Rh­(I)­(CO)_2_ species (d→π*).^62,63^ In the presence of CO, this peak grew in intensity, suggesting that Rh­(I)–CO species were present under all CO pressures. Interestingly, at 1 bar CO, a new absorption peak appeared at 550 nm and increased in intensity at 5 bar CO, which is consistent with the visual observation of the purple-red Rh species. A possible explanation for this feature is an intermolecular Rh­(I)–Rh­(I) interaction giving rise to a metal–metal transition (dσ*→pσ).^64^ This behavior has been reported for aggregates of Rh­(I) complexes that are formed through hydrophobic interactions, as well as Rh­(I) oligomers with bridging ligands.^65–68^

The same spectral features were observed when styrene was replaced with a non-coordinating solvent such as ethylbenzene, and when OTf^–^ was replaced with OAc^–^ and H_2_PO_4_^–^ (Figure S20). The wavelengths of the MLCT and metal–metal transitions did not shift across these substitutions, despite differences in donor strength and preferred binding mode among these anions. These data suggest that neither styrene nor these anions are bound in the resulting Rh species. By contrast, replacing OTf^–^ with Cl^–^ eliminated the feature at 550 nm and the MLCT at 330 nm diminished with increasing CO pressure (Figure S20). Cl^–^ is a competent ligand for Rh, as evidenced by its role in bridging Rh centers in the Rh_2_Cl_2_(CO)_4_ precatalyst. It could therefore inhibit the transformation if the transformation requires loss of Cl^–^. When TBA^+^ was replaced with K^+^, we also observed attenuation of the MLCT and no feature at 550 nm, suggesting that TBA^+^ is essential for this transformation. The quaternary nitrogen in TBA^+^ possesses neither a lone pair to donate to Rh nor any low-lying orbital to accept electron density. Direct coordination to Rh is therefore unlikely. Instead, TBA^+^ could act as a countercation for an anionic complex or solubilize aggregates of Rh­(I) complexes. Our ATR-FTIR data did not support the existence of anionic Rh carbonyl clusters that can be formed from Rh_2_Cl_2_(CO)_4_ (vide supra). We therefore propose that TBA^+^ stabilizes aggregates of Rh­(I) complexes in the styrene phase.^69–71^

In conclusion, we observed that the Rh_2_Cl_2_(CO)_4_ precatalyst undergoes various chemical reactions in the presence of CO. In one pathway, Rh_2_Cl_2_(CO)_4_ reacted with CO and H_2_O to form Rh_6_(CO)_16_, which precipitated from the electrolyte and was identified by XRD at both 1 and 5 bar CO. Control experiments indicated that Rh_6_(CO)_16_ is a minor contributor to 2-phenylpropanal formation. In another pathway, Rh_2_Cl_2_(CO)_4_ underwent CO-dependent speciation to form a purple-red species in the styrene phase. ATR-FTIR indicated that only terminal carbonyls were present, including a new feature assigned to CO bound to an electron-rich Rh center, which is corroborated by UV-Vis data supporting the presence of a Rh­(I)–CO MLCT. UV-Vis also indicated a feature at 550 nm that we propose to be a metal–metal transition from Rh­(I)–Rh­(I) interactions within aggregates of Rh­(I) complexes stabilized by TBA^+^. These pathways are also consistent with the observation of a non-integer rate order of the Rh_2_Cl_2_(CO)_4_ precatalyst (Section 2.1).

### 2.5 Saturation Behavior Suggests the Formation of a Pre-Equilibrium Intermediate

To explore the interdependence between reactants and potential in the mechanism of electro-HFN, the dependence of rate on substrate concentration was measured at low and high overpotentials (−0.76 and −1.11 V) (Figure 6A and B), while Tafel analysis was conducted at low and high CO pressures (2 and 5 bar) or low and high styrene concentrations (0.065 and 0.52 M) (Figure 6C).

**Figure 6:**
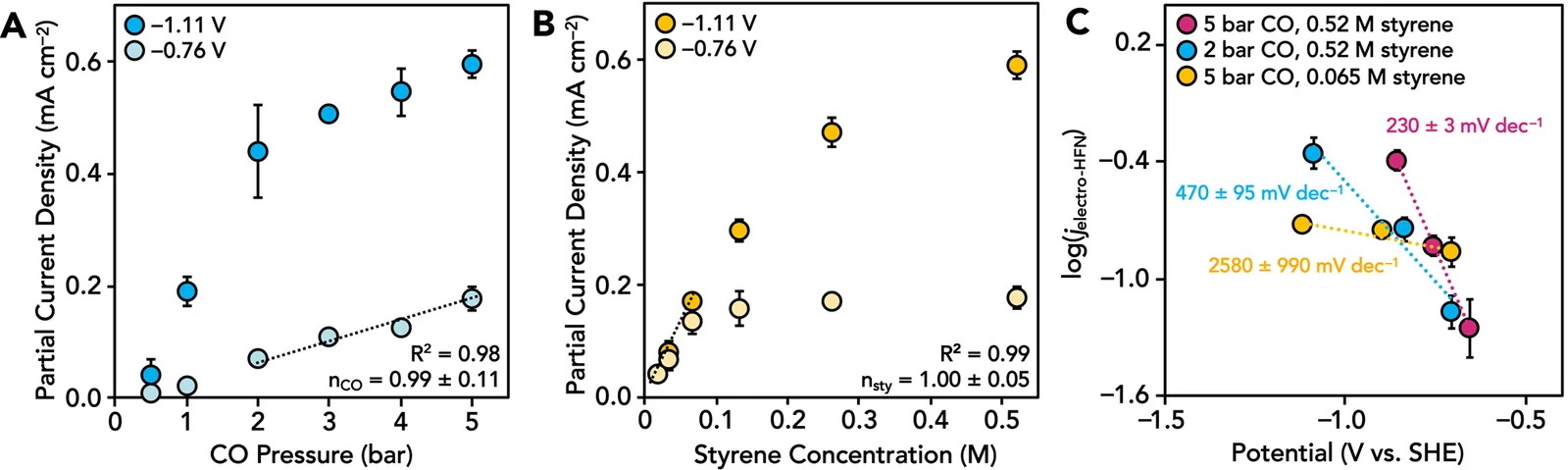
Substrate dependence experiments and Tafel data. The uncertainty of the CO and styrene rate orders (n_CO_, n_sty_) represents the standard error of the slope of a log–log plot. **A**) CO pressure dependence at −0.76 V and −1.11 V. **B**) Styrene concentration dependence at −0.76 V and −1.11 V. **C**) Potential dependence of partial current density (*j*_electro‑HFN_) at 5 bar CO and 0.52 M styrene (pink), 2 bar CO and 0.52 M styrene (blue), and 5 bar CO and 0.065 M styrene (yellow). Error bars represent one standard deviation with n ≥ 2. The uncertainty of the Tafel slopes represents the standard error of the slope. Experimental conditions unless stated otherwise: 3 mM Rh_2_Cl_2_(CO)_4_, combined styrene and ethylbenzene concentration of 0.52 M, 0.1 M TBAOTf, 50/50 v/v IPA/H_2_O, Toray 120 (5% Teflon) carbon paper cathode, 5 bar CO.

Across CO pressures of 0.5 to 5 bar, we observed a monotonic increase in partial current density with CO pressure at both low and high overpotentials (Figure 6A). CO pressures less than 2 bar were not included for rate order determination due to the formation of Rh deposits on the cathode after electrolysis (Section 2.1). At low overpotential, we extracted a CO rate order of 0.99 ± 0.11. However, at high overpotential, the CO dependence began to plateau, indicating saturation.

To measure the styrene dependence while maintaining a phase-separated electrolyte, we used ethylbenzene as a diluent, achieving a combined styrene and ethylbenzene concentration of 0.52 M. For kinetic analysis, activity is a more meaningful descriptor than concentration, but concentration can be used as a proxy for activity if the activity coefficient is near unity.^72^ We calculated the activity coefficients for styrene and ethylbenzene in a binary mixture and found them to be near unity across the entire mole fraction range (Figure S21). Across styrene concentrations of 0.016 to 0.52 M, partial current density increased with concentration before plateauing (Figure 6B). At high overpotential, a rate order of 1.00 ± 0.05 was extracted between 0.016 and 0.065 M, with the partial current density beginning to plateau at 0.13 M. However, at low overpotential, the trace began to plateau earlier, at 0.065 M, and the reaction became zero-order beyond 0.13 M.

To determine the reaction sensitivity to applied potential, we measured the partial current density from −0.66 to −1.56 V at standard conditions of 5 bar CO and 0.52 M styrene (Figure 6C, pink trace). Selecting −0.66 to −0.86 V as the linear region, we obtained a Tafel slope of 230 ± 3 mV decade^–1^, indicating moderate potential dependence. Beyond this region, the partial current density plateaued, which is best explained as a kinetic limitation from a thermochemical step because improving mass transport through stir speed had no effect on the partial current density (Figure S22 and Supplemental Discussion 4). This analysis was repeated at substrate-limited conditions. At 2 bar CO, the calculated Tafel slope was about twice as large at 470 ± 95 mV decade^–1^ (Figure 6C, blue trace). The reduced potential sensitivity, combined with the first-order CO dependence at low overpotential, suggests that a step involving CO becomes partially rate-limiting when CO is scarce. At 0.065 M styrene, the calculated Tafel slope was dramatically larger at 2580 ± 990 mV decade^–1^, indicating complete potential insensitivity in a styrene-limited regime (Figure 6C, yellow trace).

Taken together, Tafel slope analysis and substrate dependencies revealed that at low styrene concentration the reaction was insensitive to applied potential, while at high styrene concentration electron transfer (ET) became kinetically relevant. Similarly, at low overpotential, styrene saturation occurred at a lower concentration than at high overpotential. This would be expected if styrene coordination and ET both populate a pre-equilibrium intermediate, where slower ET requires less styrene to reach saturation. On the other hand, CO did not exhibit this relationship. At low overpotential, CO stayed first order across the entire pressure range, implying that CO is involved before or during the rate-determining step (RDS). This observation could be consistent with the changes in catalyst speciation we observed in the presence of CO before electrolysis. At high overpotential and substrate concentration, ET, CO, and styrene all exhibited saturation behavior, suggesting that they are involved in a pre-equilibrium before the RDS. However, the internal order of these steps cannot be resolved.

### 2.6 Rate-Determining Step Varies Across Substrate Concentration and Overpotential Regimes

To probe how the rate of ET affects the kinetic relevance of Rh–H bond cleavage, we conducted kinetic isotope effect (KIE) experiments at three potentials across high and low styrene concentrations with deuterated solvent. KIE values were calculated as the reaction rate with IPA and H_2_O (Rate_H_) divided by the reaction rate with IPA-*d*_8_ and D_2_O (Rate_D_). At high styrene concentrations (0.52 M), KIE increased with overpotential. At −0.76 V, the KIE was 1.4 ± 0.5, which is consistent with no primary KIE (Figure 7A). However, at −1.11 and −1.46 V, we calculated KIEs of 1.9 ± 0.1 and 2.3 ± 0.1, respectively, consistent with a primary KIE. The increase in KIE with increasing overpotential suggests that as ET becomes more facile, Rh–H bond cleavage becomes increasingly rate-limiting. This trend was also observed in the prior kinetic analysis using Rh@CeO_2_ as the electro-HFN catalyst, albeit with lower KIE magnitudes here.^5^ On the other hand, at low styrene concentrations (0.065 M), we calculated KIEs between 0.9 ± 0.1 and 1.4 ± 0.3, which are consistent with no primary KIE.

**Figure 7:**
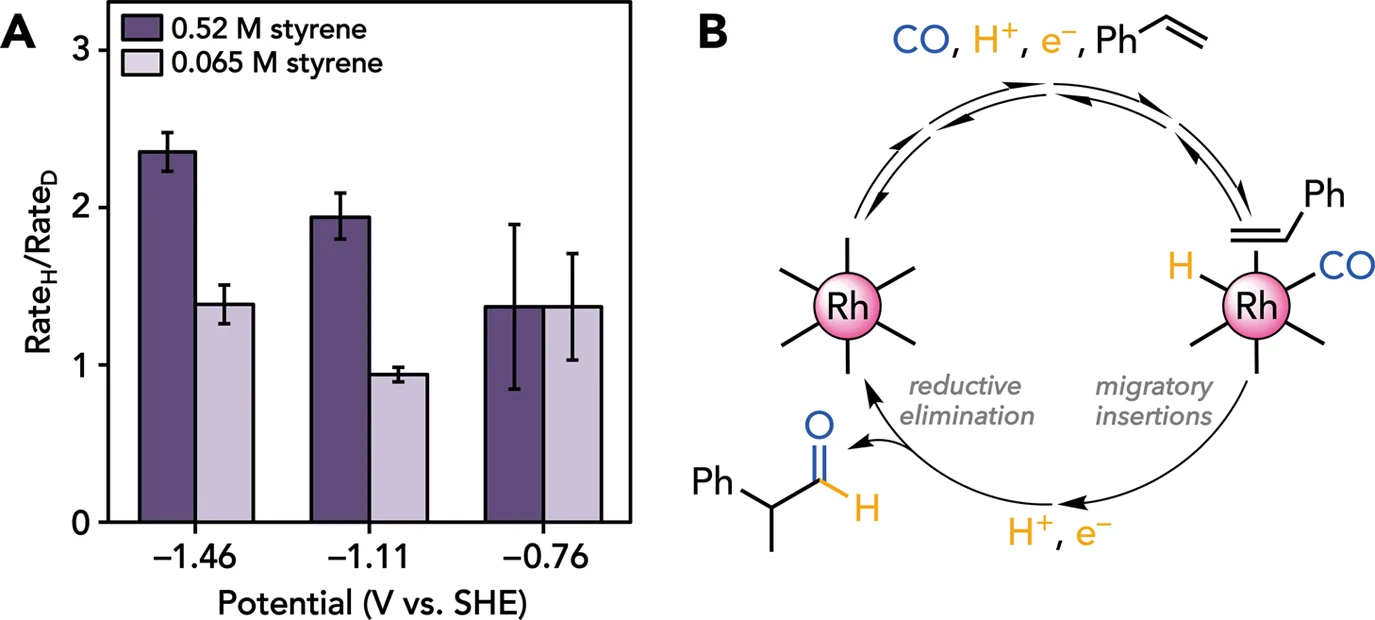
Kinetic isotope experiments and a proposed mechanism for electro-HFN. **A**) Potential dependence of KIE (Rate_H_/Rate_D_). Error bars represent one standard deviation with n ≥ 2. **B**) Proposed mechanistic pathway consistent with kinetic studies. Experimental conditions unless stated otherwise: 3 mM Rh_2_Cl_2_(CO)_4_, combined styrene and ethylbenzene concentration of 0.52 M, 0.1 M TBAOTf, 50/50 v/v IPA/H_2_O, Toray 120 (5% Teflon) carbon paper cathode, 5 bar CO.

To briefly probe the proton dependence of this reaction, we varied HOTf concentration from 1 to 15 mM at −1.11 V vs. SHE and found a weak proton order of 0.13 ± 0.01 (Figure S23). This observation is also consistent with the electro-HFN system using Rh@CeO_2_, where little to no pH dependence was observed at HOTf concentrations less than 20 mM.^5^ These data suggest that the KIE did not originate from a simple proton transfer (PT), but more likely arose from a migratory insertion or reductive elimination step involving a hydride.

Taken together, the substrate, potential, and KIE data support a mechanism in which the RDS switches depending on the substrate concentration and overpotential regime. In the first regime (low styrene concentration, all overpotentials), no significant primary KIE was observed, and the reaction was insensitive to applied potential. Given the first-order dependence on styrene and the precedent for olefin coordination as the RDS in the thermo-HFN literature, styrene coordination was the most likely RDS in this regime.^73^ In the second regime (high styrene concentration, low overpotential), no significant primary KIE was observed. Since the styrene rate order was zero under these conditions, styrene coordination was excluded as the RDS. By elimination, ET is the most likely remaining RDS. In the third regime (high styrene concentration, high overpotential), a significant primary KIE was observed, suggesting the RDS involved cleavage of a Rh–H bond. Because proton dependence was weak under these conditions, the RDS was unlikely to be simple PT to form Rh–H. Migratory insertion of the hydride into the olefin or reductive elimination of the product is more likely to be the RDS. These findings are consistent with a proposed reaction pathway in which CO binding, PT, ET, and styrene coordination form a pre-equilibrium intermediate. Migratory insertions and reductive elimination then generate 2-phenylpropanal (Figure 7B; expanded cycle shown in Figure S24).

## 3 Conclusions

This work reports an electro-HFN system catalyzed by homogeneous Rh that achieved a partial current density and FE of 1.1 ± 0.1 mA cm^–2^ and 29 ± 1%, respectively. Exploration of reaction conditions highlighted the importance of hydrophobicity, which was independently tuned through the choice of cathode, organic cosolvent, and supporting electrolyte salt. Rh_2_Cl_2_(CO)_4_ was identified as a commercially available precatalyst that achieved the highest partial current density for electro-HFN. In the presence of CO, Rh_2_Cl_2_(CO)_4_ undergoes CO-dependent speciation, as evidenced by the changes in ATR-FTIR and UV-Vis spectra, which suggest the formation of Rh­(I)–CO species. Kinetic experiments revealed a complex mechanism in which steps involving ET, substrate coordination, and Rh–H bond cleavage are rate-limiting under various substrate concentration and overpotential regimes.

Looking forward, the insights of this work can be applied to the design of an electro-HFN system with enhanced selectivity and rate by further tuning the hydrophobicity of the reaction medium and the coordination environment of the precatalyst. Additionally, this work demonstrated the susceptibility of soluble Rh catalysts to electrodeposition onto the cathode. We mitigated Rh electrodeposition by employing higher CO pressures and biphasic electrolytes. However, future efforts in homogeneous electro-HFN catalysis should prioritize the design of robust Rh complexes that are able to operate at ambient pressures in a single-phase electrolyte. Beyond electro-HFN, these guiding principles can be applied to other reductive C–C bond formation reactions that seek to transform nonpolar substrates under polar electrochemical conditions.

## 4 Experimental Section

### 4.1 Anode Preparation

The procedure to make ruthenium iridium oxide on titanium (RuIrO_*x*_/Ti) was adapted from literature preparations.^74,75^ High-porosity Ti felt (Fuel Cell Store) was punched into 16 mm disks and sonicated in a 10% nitric acid (70% solution in water, Thermo Fisher Scientific) bath for 15 min, rinsed with water, and air-dried. A solution of 5 mM iridium chloride hydrate (99.9%, Sigma-Aldrich) and 5 mM ruthenium chloride hydrate (technical grade, Sigma-Aldrich) was prepared in a 50/50 v/v IPA/H_2_O solution. On a hot plate set to 80 °C, 150 μL of the iridium and ruthenium chloride solution was drop cast onto the Ti felt disks in 30 μL aliquots. The Ti disks were dried completely between aliquots. Finally, the Ti felt disks were calcined in a muffle furnace at 450 °C for 4 h at a ramp rate of 10 °C min^–1^.

### 4.2 Cathode Preparation

CP (Toray 120, Fuel Cell Store) sheets were punched into 14 mm disks before use. GC (99.9%, MSE Supplies) and Pt (99.99%, Beantown Chemical), Rh (99.9%, Sigma-Aldrich), and Au (99.95%, Beantown Chemical) foils were rinsed with water and acetone (99.5%, VWR) before use. Ag (99.95%, Beantown Chemical), Ti (99.99%, Beantown Chemical), and Sn (99.5%, Beantown Chemical) foils were mechanically polished with 600-grit silicon carbide sandpaper (MSE Supplies, GSC-P600) and rinsed with water and acetone before use.

### 4.3 Supporting Electrolyte Salt Preparation

TEAOTf and TPAOTf salts were prepared by titrating a solution of 1 M HOTf (99%, Sigma-Aldrich) with tetraethylammonium hydroxide (35 wt % in H_2_O, Sigma-Aldrich) or tetrapropylammonium hydroxide (1.0 M solution in H_2_O, Sigma-Aldrich). A pH meter was submerged in the solution and the pH was continuously monitored until it was near 7. Through this method, 0.25 M solutions of each salt were prepared in a 50/50 v/v IPA/H_2_O solution.

### 4.4 Electrolyte Preparation

The electrolyte used in the majority of experiments was composed of 0.52 M styrene (99%, Sigma-Aldrich) and 0.1 M TBAOTf (99%, Sigma-Aldrich) in a 50/50 v/v IPA/H_2_O solution to make up a total electrolyte volume of 3.4 mL. Molarities were calculated using the total volume of the mixture. The resulting phase-separated electrolyte was continuously stirred at 800 rpm during electrochemical experiments. Based on the solubility of each Rh complex, the complexes were dissolved in either the styrene phase or IPA/H_2_O phase before they were added to the electrochemical cell. Rh_2_Cl_2_(CO)_4_ (97%, Sigma-Aldrich), Rh_2_Cl_2_(COE)_4_ (98%, Sigma-Aldrich), Rh_2_Cl_2_(COD)_2_ (98%, Sigma-Aldrich), and Rh­(acac)_3_ (97%, Sigma-Aldrich) were soluble in the styrene phase. RhCl_3_·xH_2_O (99.99%, Beantown Chemical), Rh­(NO_3_)_3_·xH_2_O (36% Rh basis, Sigma-Aldrich), Rh_2_(OAc)_4_ (99.9%, Sigma-Aldrich), and Rh_2_(SO_4_)_3_ (8% in H_2_O, Sigma-Aldrich) were soluble in the IPA/H_2_O phase. With the exception of RhCl_3_·xH_2_O, all precatalysts were immediately soluble upon addition to either styrene or the IPA/H_2_O solution. RhCl_3_·xH_2_O was sonicated in the aqueous electrolyte for 5 min to disperse the solid, followed by stirring at 90 °C for 5 min. Notably, RhCl_3_·xH_2_O in the standard electrolyte began as a red powder suspension and became a pale yellow solution upon heating.

### 4.5 Electrochemical Experiment Setup

Electrochemical experiments were performed in custom-machined PEEK sandwich cells (Figure S1). Back plates were lined with aluminum tape (76925A107, McMaster-Carr) to serve as current collectors. Electrolyte chambers possessed grooves for chemically resistant Viton O-rings (1170N32/1170N26, McMaster-Carr). The cell was held together by 2″ stainless steel screws and wingnuts (92185A207/90866A009, McMaster-Carr). Due to the porosity of the RuIrO_*x*_ anodes, a fluorosilicone rubber gasket (2183T11, McMaster-Carr) and Ti foil were added to make contact between the anode and the aluminum tape. When porous cathodes were used, a glassy carbon plate (SU6009, MSE Supplies) was added to make contact between the cathode and the aluminum tape. An Ag/AgCl leakless electrode (ET072-1, MSE Supplies) was used as the reference electrode. A 3 × 10 mm magnetic stir bar (22–271783, Fisher Scientific) was added to the electrolyte chamber to achieve convection during the reaction.

Once the cell was assembled and the electrolyte was confirmed to be well mixed at 800 rpm, 1 bar CO was bubbled through the stirring electrolyte for 5 min at 10 sccm using a mass flow controller (MFC). The cell was plugged and pressurized to 5 bar at 10 sccm. Then, the cell was left to equilibrate for 5 min prior to electrolysis. For chronoamperometry experiments, the solution resistance was measured by electrochemical impedance spectroscopy. For reaction condition screening, a 70% automatic *iR* correction was used. For kinetic experiments, an 85% manual *iR* correction was used. The remaining 30% or 15% of the *iR* drop was subtracted from the applied potential using the average total current measured during the electrolysis. An electrolysis was typically run for 30 min except for experiments screening cathodes (Figure 2B, 15 min), solvents (Figure 2C, 1 h), and supporting electrolyte salts (Figure 2D, 1 h). After electrolysis, the cell was disassembled, and the cell parts were rinsed with acetone.

### 4.6 Product Quantification via Gas Chromatography–Mass Spectrometry

To quantify 2-phenylpropanal and ethylbenzene, we used a GC-MS instrument fitted with a DB-WAX column in split-injection mode (Figure S2). After electrolysis, the electrolyte was pipetted into a 20 mL scintillation vial. 1 mL of acetonitrile (99.5%, VWR) was added to the cell to rinse the remaining electrolyte and then transferred to the 20 mL scintillation vial. 1 mL of Milli-Q water was added to the cell to rinse the remaining electrolyte and then transferred to the 20 mL scintillation vial. Then, 20 μL of 0.16 M 1,3,5-trimethoxybenzene (TMB) (99%, Sigma-Aldrich) and 1.5 mL of hexanes (99.9%, VWR) were added to the 20 mL scintillation vial. The vial was capped and vigorously vortexed for 15 seconds. The mixture was allowed to settle for several minutes, and the hexanes fraction was collected into a 1.5 mL GC-MS sample vial.

The temperature sequence was programmed to begin at 40 °C for 1 min, then ramp to 250 °C at 20 °C min^–1^ and hold at 250 °C for 6 min. A 6 min solvent delay was included in the sequence to avoid saturating the detector with the excess styrene in the sample. To quantify ethylbenzene, the solvent delay was decreased to 2.5 min. We generated standard curves with known amounts of 2-phenylpropanal (98%, Sigma-Aldrich) and ethylbenzene (99%, Sigma-Aldrich) using the ratio of their base ion peak areas (*m/z* 105 and 91) relative to the molecular ion peak area of TMB (*m/z* 168) (Figures S3 and S4). The FE and partial current density were calculated using the moles of product detected by GC-MS and the geometric surface area (1 cm^2^) (eqs S1 and S2).

### 4.7 Product Quantification via Nuclear Magnetic Resonance Spectroscopy

To quantify 2-phenylpropanal, we ran ^1^H NMR spectroscopy experiments on a Bruker 400 MHz spectrometer (Figure S5). After electrolysis, 200 μL of an aqueous 0.4 M formic acid (95%, Sigma-Aldrich) solution was added to the electrolyte while the electrolyte was stirring. Then, the electrolyte was pipetted into a 20 mL scintillation vial. The cell was rinsed with 700 μL of acetone and the rinse was added to the 20 mL scintillation vial. Notably, the electrolyte became a single phase upon addition of acetone. For solvent and supporting electrolyte salt screening, the volume of acetone was increased to 1.4 mL to ensure that a single-phase solution was achieved. Finally, NMR samples were prepared by adding 200 μL of the single-phase electrolyte solution to 400 μL of dimethyl sulfoxide-*d*_6_ (99.9%, Cambridge Isotope Laboratories). The FE and partial current density were calculated using the moles of product detected by NMR and the geometric surface area (1 cm^2^) (eqs S1 and S2).

### 4.8 H_2_ Quantification via Gas Chromatography with a Thermal Conductivity Detector

To quantify H_2_, we used an SRI 8610C GC-TCD instrument fitted with a molecular-sieve column (Figure S6). Argon was used as the carrier gas. First, we generated a standard curve of known H_2_ concentrations by changing the relative flow rates of the H_2_ and N_2_ streams while maintaining a total flow rate of 60 sccm through the 1 mL sample loop (Figure S7). The temperature sequence was programmed to begin at 40 °C for 2 min, then ramped to 150 °C at 50 °C min^–1^ and held at 150 °C for 4 min.

To quantify H_2_ during an electrolysis, a back pressure regulator (Zaiput BPR-10) was plumbed directly downstream of the electrochemical cell to maintain a pressure of 5 bar CO in the reactor headspace during flow. To achieve a flow rate of 3 sccm through the reactor and into the sample loop, the BPR pressure was set to 4.9 bar. To increase the detected peak area of H_2_ at low potentials, the flow rate was lowered to 1 and 2 sccm at −0.66 and −0.76 V vs. SHE, respectively.

After the typical pressurization procedure detailed in Section 4.5 was complete, the MFC was set to the desired flow rate (1–3 sccm). For a typical 30 min electrolysis, the GC-TCD sample sequence was initiated at 15 and 30 min into the electrolysis. The H_2_ peak areas were averaged between these time points. The FE of H_2_ was calculated using the total flow rate, % H_2_, total charge passed, and length of the electrolysis (eq S3).

### 4.9 Powder X-ray Diffraction

Powder XRD experiments were performed using a Rigaku SmartLab diffractometer (Cu K*α*). XRD scans were collected from 5° to 65° using a 10° min^–1^ scan rate. Samples were prepared by pressurization of the standard electrolyte at 1 and 5 bar CO for 30 min, followed by centrifugation at 19,090 rcf for 5 min. The supernatant was decanted and the precipitate was air-dried overnight.

### 4.10 Attenuated Total Reflectance Fourier Transform Infrared Absorption Spectroscopy

Ex situ ATR-FTIR spectroscopy measurements were performed using a Nicolet iS50 Fourier transform infrared spectrometer. To measure the sample at 1 bar CO, CO was bubbled through the electrolyte in the electrochemical cell for 30 min while stirring. To measure the sample at 5 bar CO, CO was bubbled through the electrolyte in the electrochemical cell for 5 min and then the cell was pressurized to 5 bar CO and left for 30 min. Then, the cell was depressurized and 1 mL of the sample was centrifuged at 19,090 rcf for 5 min to achieve complete phase separation and removal of the Rh_6_(CO)_16_ precipitate. Finally, 40 μL of the organic phase was dropped onto a silicon crystal for measurement. The background for each sample was an identically prepared electrolyte without Rh precatalyst that had undergone the same CO and centrifugation treatment.

### 4.11 UV-Visible Spectroscopy

Ex situ UV-Vis spectroscopy measurements were performed using an Agilent Cary 60 UV-Vis spectrophotometer. A reference spectrum was collected on a sample of the standard electrolyte without the precatalyst and subtracted from the sample spectrum. To measure the sample at 1 bar CO, CO was bubbled through the electrolyte in the electrochemical cell for 30 min. To measure the sample at 5 bar CO, CO was bubbled through the electrolyte in the electrochemical cell for 5 min and then the cell was pressurized to 5 bar CO for 30 min. Then, the cell was depressurized and 1 mL of the sample was centrifuged at 19,090 rcf for 5 min to achieve complete phase separation and removal of the Rh_6_(CO)_16_ precipitate. Finally, 20 μL of the organic phase was diluted 250-fold with IPA and measured in a quartz cuvette.

## Supplementary Material



## Notes

Reagent information, experimental protocols, supplemental data, and discussions (PDF)
