# Supplementary material for: Electrochemical Hydroformylation with a Homogeneous Rhodium Catalyst
Source: J Am Chem Soc. 2026 Oct 1;148(40):43754–66. doi: 10.1021/jacs.6c15582 (PMC13674260; doi:10.1021/jacs.6c15582)
Supplement: Supplementary file 1 [file ja6c15582_si_001.pdf]

Electrochemical Hydroformylation with a Homogeneous Rhodium Catalyst

Emma L. Cosner,[1] Spencer P. Delgado-Kukuczka,[1] Haochen Zhang,[1] Abigail L. Blenko,[1] Evan V. Miu,[1] Karthish Manthiram[1,*]

[1]Division of Chemistry and Chemical Engineering, California Institute of Technology, Pasadena, California 91125, United States

*Correspondence: karthish@caltech.edu

Table of Contents

1. Materials and Methods 3
1.1 Materials and Equipment 3
1.2 Anode Preparation 5
1.3 Cathode Preparation 5
1.4 Supporting Electrolyte Salt Preparation 5
1.5 Electrolyte Preparation 5
1.6 Electrochemical Experiment Setup 6
Figure S1. Electrochemical cell parts 6
1.7 Product Quantification via Gas Chromatography–Mass Spectrometry 7
Figure S2. Example gas chromatogram 7
Figure S3. Mass spectra of 1,3,5-trimethoxybenzene, ethylbenzene, 2-phenylpropanal 8
Figure S4. Standard curves of 2-phenylpropanal and ethylbenzene 8
Equation S1. FE calculation for GC-MS and NMR quantification 9
Equation S2. Partial current density calculation for GC-MS and NMR quantification 9
1.8 Product Quantification via Nuclear Magnetic Resonance Spectroscopy 10
Figure S5. Example ^{1}H NMR of 2-phenylpropanal, 3-phenylpropanal, and formic acid 10
1.9 H_2 Quantification via Gas Chromatography with a Thermal Conductivity Detector 11
Figure S6. Example TCD chromatogram of H_2 and CO 11
Figure S7. Standard curve of H_2 concentration 11
Equation S3. FE calculation for GC-TCD 12
1.10 Powder X-ray Diffraction 13
1.11 Attenuated Total Reflectance Fourier Transform Infrared Absorption Spectroscopy 13
1.12 UV-visible Spectroscopy 13
1.13 X-ray Photoelectron Spectroscopy 13
1.14 Scanning Electron Microscopy and Energy-Dispersive X-ray Spectroscopy 14
2. Supplemental Data and Discussions 15
Figure S8. Partial current density of Rh complexes normalized by Rh moles 15
Supplemental Discussion 1. Explanation of Rh plating behavior among complexes 15
Figure S9. Schematic of conditions resulting in Rh deposits on cathode 16

Figure S10. Scanning electron microscopy of Rh deposits on cathode from $RhCl_3$............... 16
Figure S11. Scanning electron microscopy of Rh deposits on cathode from $Rh_2Cl_2(CO)_4$.... 17
Figure S12. X-ray photoelectron spectroscopy of Rh deposits on cathode............................ 17
Figure S13. Control experiments with Rh deposits.. 18
Figure S14. $Rh_2Cl_2(CO)_4$ concentration dependence... 18
Supplemental Discussion 2. Capacitance measurements as cathode ECSA estimates.......... 19
Equation S4. Double layer capacitance.. 19
Equation S5. Electrochemically active surface area... 19
Figure S15. Partial current normalization by ECSA... 20
Figure S16. Tetrabutylammonium concentration dependence... 20
Figure S17. Cyclic voltammogram of $Rh_2Cl_2(CO)_4$.. 21
Supplemental Discussion 3. Local H_2 concentration in control experiments........................ 22
Equation S6. Maximum interfacial H_2 concentration calculation... 22
Figure S18. Average total current density as a function of styrene concentration................. 23
Figure S19. Color changes of $Rh_2Cl_2(CO)_4$ in the electrolyte and at 1 and 5 bar CO............. 23
Figure S20. UV-visible spectroscopy of various electrolyte conditions in CO....................... 24
Figure S21. UNIFAC model of styrene and ethylbenzene activity coefficients..................... 25
Figure S22. Partial current density stir rate dependence... 25
Supplemental Discussion 4. Maximum transport-limited current density............................ 26
Equation S7. Maximum transport-limited current density of CO... 26
Figure S23. Partial current density HOTf concentration dependence.................................... 27
Figure S24. Possible mechanism for electro-HFN... 28
3. References... 29

1. Materials and Methods

1.1 Materials and Equipment

All chemicals and materials were used as received unless otherwise specified.

Complexes

- Iridium(III) chloride hydrate, 99.9%, Sigma-Aldrich
- Ruthenium(III) chloride hydrate, technical grade, Sigma-Aldrich
- Di-μ-chloro-tetracarbonyldirhodium(I) ($Rh_2Cl_2(CO)_4$), 97%, Sigma-Aldrich
- Chloro(1,5-cyclooctadiene)rhodium(I) dimer ($Rh_2Cl_2(COD)_2$), 98%, Sigma-Aldrich
- Chlorobis(cyclooctene)rhodium(I) dimer ($Rh_2Cl_2(COE)_4$), 98%, Sigma-Aldrich
- Rhodium(III) chloride hydrate ($RhCl_3 \cdot xH_2O$), 99.99%, Beantown Chemical
- Rhodium(III) nitrate hydrate ($Rh(NO_3)_3 \cdot xH_2O$), 36% Rh basis, Sigma-Aldrich
- Rhodium(III) acetylacetonate ($Rh(acac)_3$), 97%, Sigma-Aldrich
- Rhodium(II) acetate dimer ($Rh_2(OAc)_4$), 99.9%, Sigma-Aldrich
- Rhodium(III) sulfate solution ($Rh_2(SO_4)_3$), 8% in H_2O, Sigma-Aldrich
- Hexarhodium(0) hexadecacarbonyl, 98%, Thermo Fisher Scientific

Electrodes

- High-porosity titanium fiber felt, Fuel Cell Store
- Carbon paper, Toray Carbon Paper 120, 5% Teflon, Fuel Cell Store
- Glassy carbon, 2 mm thick, 99.9%, MSE Supplies
- Gold foil, 0.025 mm thick, 99.95% trace metals basis, Beantown Chemical
- Silver foil, 0.5 mm thick, 99.95% trace metals basis, Beantown Chemical
- Tin foil, 0.25 mm thick, 99.5% trace metals basis, Beantown Chemical
- Rhodium foil, 0.1 mm thick, 99.9% trace metals basis, Sigma-Aldrich
- Platinum foil, 0.025 mm thick, 99.99% trace metals basis, Beantown Chemical
- Titanium foil, 0.25 mm thick, 99.99% trace metals basis, Beantown Chemical

Solvents

- Dimethyl sulfoxide-d_6, 99.9%, Cambridge Isotope Laboratories
- Isopropanol-d_8, 99%, Cambridge Isotope Laboratories
- Deuterium oxide, 99.9%, Cambridge Isotope Laboratories
- Milli-Q® water, 18.2 MΩ cm, Merck, Millipore Corporation
- Isopropanol, ≥99.5% ACS, VWR
- Tetrahydrofuran, ≥99% stabilized ACS, VWR
- Acetonitrile, ≥99.5% ACS, VWR
- Ethanol, 99.5% ACS, VWR
- Hexanes, 99.9% ACS, VWR
- Acetone, 99.5% ACS, VWR

Gases

- Carbon Monoxide Research Grade Size 200, 99.999%, Airgas
- Hydrogen, Ultra High Purity, 99.999%, Airgas
- 50% Carbon Monoxide, 50% Hydrogen, Size 35, Airgas
- Nitrogen, 99.9%, Airgas
- Argon, 99.9%, Airgas

Electrolyte Components

- Tetrabutylammonium trifluoromethanesulfonate, 99.0%, Sigma-Aldrich
- Tetrabutylammonium acetate, 97%, Sigma-Aldrich
- Tetrabutylammonium phosphate monobasic solution, 1 M in H_2O, Sigma-Aldrich
- Tetrabutylammonium chloride, 97%, Sigma-Aldrich
- Tetraethylammonium hydroxide solution, 35 wt. % in H_2O, Sigma-Aldrich
- Tetrapropylammonium hydroxide solution, 1.0 M in H_2O, Sigma-Aldrich
- Potassium trifluoromethanesulfonate, 98%, Sigma-Aldrich
- Formic acid, reagent grade, 95%, Sigma-Aldrich
- Trifluoromethanesulfonic acid, ReagentPlus®, 99%, Sigma-Aldrich
- Styrene, 99%, ReagentPlus®, contains 4-tert-butylcatechol as stabilizer, Sigma-Aldrich
- 2-Phenylpropionaldehyde, 98%, Sigma-Aldrich
- 3-Phenylpropionaldehyde, 98%, Sigma-Aldrich
- Ethylbenzene, ReagentPlus®, 99%, Sigma-Aldrich
- 1,3,5-Trimethoxybenzene, ReagentPlus®, 99%, Sigma-Aldrich
- Nitric acid, 70% solution in water, Thermo Fisher Scientific

Cell Components

- Leakless Miniature Ag/AgCl Reference Electrode, MSE Supplies, eDAQ
- Bel-Art™ SP Scienceware™ Flea Micro Spinbar™ Magnetic Stir Bars, 3 × 10 mm
- Chemical-Resistant Low-Temperature Fluorosilicone Rubber, McMaster-Carr
- Super-Corrosion-Rst 316 SS Sckt Head Screw 8-32 Thread Size, 2" Long, Fully Threaded, McMaster-Carr
- Zinc-Plated Steel Wing Nut 8-32 Thread Size, 13/32" Base Diameter, McMaster-Carr
- Aluminum tape, 1" wide, McMaster-Carr
- Chemically resistant Viton O-rings, McMaster-Carr

Equipment

- Thermolyne Small Benchtop Muffle Furnace, 1.3 L, 1100 °C
- BioLogic 16 Channel Potentiostat
- Bruker 400 MHz Nuclear Magnetic Resonance Spectrometer
- SRI 8610C gas chromatograph, 5 Å molecular-sieve column, with a thermal conductivity detector
- Hewlett-Packard 5972 Gas Chromatography Mass Spectrometer
- Nicolet iS50 Fourier Transform Infrared Spectrometer
- Agilent Cary 60 UV-Vis Spectrophotometer
- Kratos AXIS Ultra spectrometer
- ZEISS 1550VP High-Resolution Analytical Scanning Electron Microscope

1.2 Anode Preparation

The procedure to make ruthenium iridium oxide on titanium ($RuIrO_x$/Ti) was adapted from literature preparations.[1,2] High-porosity Ti felt (Fuel Cell Store) was punched into 16 mm disks and sonicated in a 10% nitric acid (70% solution in water, Thermo Fisher Scientific) bath for 15 minutes, rinsed with water, and air-dried. A solution of 5 mM iridium chloride hydrate (99.9%, Sigma-Aldrich) and 5 mM ruthenium chloride hydrate (technical grade, Sigma-Aldrich) was prepared in a 50/50 v/v IPA/H_2O solution. On a hot plate set to 80 °C, 150 µL of the iridium and ruthenium chloride solution was drop cast onto the Ti felt disks in 30 µL aliquots. The Ti disks were dried completely between aliquots. Finally, the Ti felt disks were calcined in a muffle furnace at 450 °C for 4 hours at a ramp rate of 10 °C min^{-1}.

1.3 Cathode Preparation

CP (Toray 120, Fuel Cell Store) sheets were punched into 14 mm disks before use. GC (99.9%, MSE Supplies) and Pt (99.99%, Beantown Chemical), Rh (99.9%, Sigma-Aldrich), and Au (99.95%, Beantown Chemical) foils were rinsed with water and acetone (99.5%, VWR) before use. Ag (99.95%, Beantown Chemical), Ti (99.99%, Beantown Chemical), and Sn (99.5%, Beantown Chemical) foils were mechanically polished with 600-grit silicon carbide sandpaper (MSE Supplies, GSC-P600) and rinsed with water and acetone before use.

1.4 Supporting Electrolyte Salt Preparation

TEAOTf and TPAOTf salts were prepared by titrating a solution of 1 M HOTf (99%, Sigma-Aldrich) with tetraethylammonium hydroxide (35 wt. % in H_2O, Sigma-Aldrich) or tetrapropylammonium hydroxide (1.0 M solution in H_2O, Sigma-Aldrich). A pH meter was submerged in the solution and the pH was continuously monitored until it was near 7. Through this method, 0.25 M solutions of each salt were prepared in a 50/50 v/v IPA/H_2O solution.

1.5 Electrolyte Preparation

The electrolyte used in the majority of experiments was composed of 0.52 M styrene (99%, Sigma-Aldrich) and 0.1 M TBAOTf (99%, Sigma-Aldrich) in a 50/50 v/v IPA/H_2O solution to make up a total electrolyte volume of 3.4 mL. Molarities were calculated using the total volume of the mixture. The resulting phase-separated electrolyte was continuously stirred at 800 rpm during electrochemical experiments. Based on the solubility of each Rh complex, the complexes were dissolved in either the styrene phase or IPA/H_2O phase before they were added to the electrochemical cell. $Rh_2Cl_2(CO)_4$ (97%, Sigma-Aldrich), $Rh_2Cl_2(COE)_4$ (98%, Sigma-Aldrich), $Rh_2Cl_2(COD)_2$ (98%, Sigma-Aldrich), and $Rh(acac)_3$ (97%, Sigma-Aldrich) were soluble in the styrene phase. $RhCl_3 \cdot xH_2O$ (99.99%, Beantown Chemical), $Rh(NO_3)_3 \cdot xH_2O$ (36% Rh basis, Sigma-Aldrich), $Rh_2(OAc)_4$ (99.9%, Sigma-Aldrich), and $Rh_2(SO_4)_3$ (8% in H_2O, Sigma-Aldrich) were soluble in the IPA/H_2O phase. With the exception of $RhCl_3 \cdot xH_2O$, all precatalysts were immediately soluble upon addition to either styrene or the IPA/H_2O solution. $RhCl_3 \cdot xH_2O$ was sonicated in the aqueous electrolyte for 5 minutes to disperse the solid, followed by stirring at 90 °C for 5 minutes. Notably, $RhCl_3 \cdot xH_2O$ in the standard electrolyte began as a red powder suspension and became a pale yellow solution upon heating.

1.6 Electrochemical Experiment Setup

Electrochemical experiments were performed in custom-machined PEEK sandwich cells (**Figure S1**). Back plates were lined with aluminum tape (76925A107, McMaster-Carr) to serve as current collectors. Electrolyte chambers possessed grooves for chemically resistant Viton O-rings (1170N32/1170N26, McMaster-Carr). The cell was held together by 2" stainless steel screws and wingnuts (92185A207/90866A009, McMaster-Carr). Due to the porosity of the $RuIrO_x$ anodes, a fluorosilicone rubber gasket (2183T11, McMaster-Carr) and Ti foil were added to make contact between the anode and the aluminum tape. When porous cathodes were used, a glassy carbon plate (SU6009, MSE Supplies) was added to make contact between the cathode and the aluminum tape. An Ag/AgCl leakless electrode (ET072-1, MSE Supplies) was used as the reference electrode. A 3 × 10 mm magnetic stir bar (22-271783, Fisher Scientific) was added to the electrolyte chamber to achieve convection during the reaction.

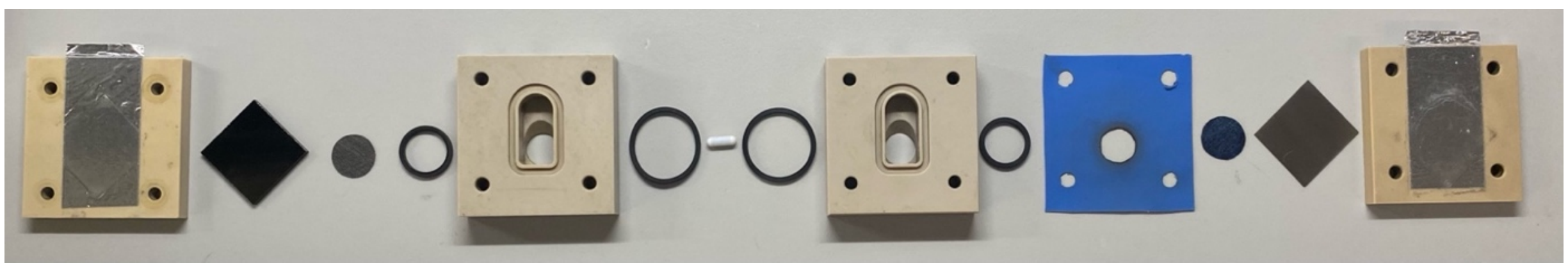

Figure S1. PEEK electrochemical cell parts laid out in assembly order.

Once the cell was assembled and the electrolyte was confirmed to be well mixed at 800 rpm, 1 bar CO was bubbled through the stirring electrolyte for 5 minutes at 10 sccm using a mass flow controller (MFC). The cell was plugged and pressurized to 5 bar at 10 sccm. Then, the cell was left to equilibrate for 5 minutes prior to electrolysis. For chronoamperometry experiments, the solution resistance was measured by electrochemical impedance spectroscopy. For reaction condition screening, a 70% automatic *iR* correction was used. For kinetic experiments, an 85% manual *iR* correction was used. The remaining 30% or 15% of the *iR* drop was subtracted from the applied potential using the average total current measured during the electrolysis. An electrolysis was typically run for 30 minutes except for experiments screening cathodes (Figure 2B, 15 minutes), solvents (Figure 2C in the main text, 1 hour), and supporting electrolyte salts (Figure 2D in the main text, 1 hour). After electrolysis, the cell was disassembled, and the cell parts were rinsed with acetone.

1.7 Product Quantification via Gas Chromatography–Mass Spectrometry

To quantify 2-phenylpropanal and ethylbenzene, we used a GC-MS instrument fitted with a DB-WAX column in split-injection mode (**Figure S2**). After electrolysis, the electrolyte was pipetted into a 20 mL scintillation vial. 1 mL of acetonitrile (99.5%, VWR) was added to the cell to rinse the remaining electrolyte and then transferred to the 20 mL scintillation vial. 1 mL of Milli-Q® water was added to the cell to rinse the remaining electrolyte and then transferred to the 20 mL scintillation vial. Then, 20 µL of 0.16 M 1,3,5-trimethoxybenzene (TMB) (99%, Sigma-Aldrich) and 1.5 mL of hexanes (99.9%, VWR) were added to the 20 mL scintillation vial. The vial was capped and vigorously vortexed for 15 seconds. The mixture was allowed to settle for several minutes, and the hexanes fraction was collected into a 1.5 mL GC-MS sample vial.

The temperature sequence was programmed to begin at 40 °C for 1 minute, then ramped to 250 °C at 20 °C min^{-1} and held at 250 °C for 6 minutes. A six-minute solvent delay was included in the sequence to avoid saturating the detector with the excess styrene in the sample. To quantify ethylbenzene, the solvent delay was decreased to 2.5 minutes. We generated standard curves with known amounts of 2-phenylpropanal (98%, Sigma-Aldrich) and ethylbenzene (99%, Sigma-Aldrich) using the ratio of their base ion peak areas (*m/z* 105 and 91) relative to the molecular ion peak area of TMB (*m/z* 168) (**Figures S3 and S4**). The FE and partial current density were calculated using the moles of product detected by GC-MS and the geometric surface area (1 cm^2) (**Equations S1 and S2**).

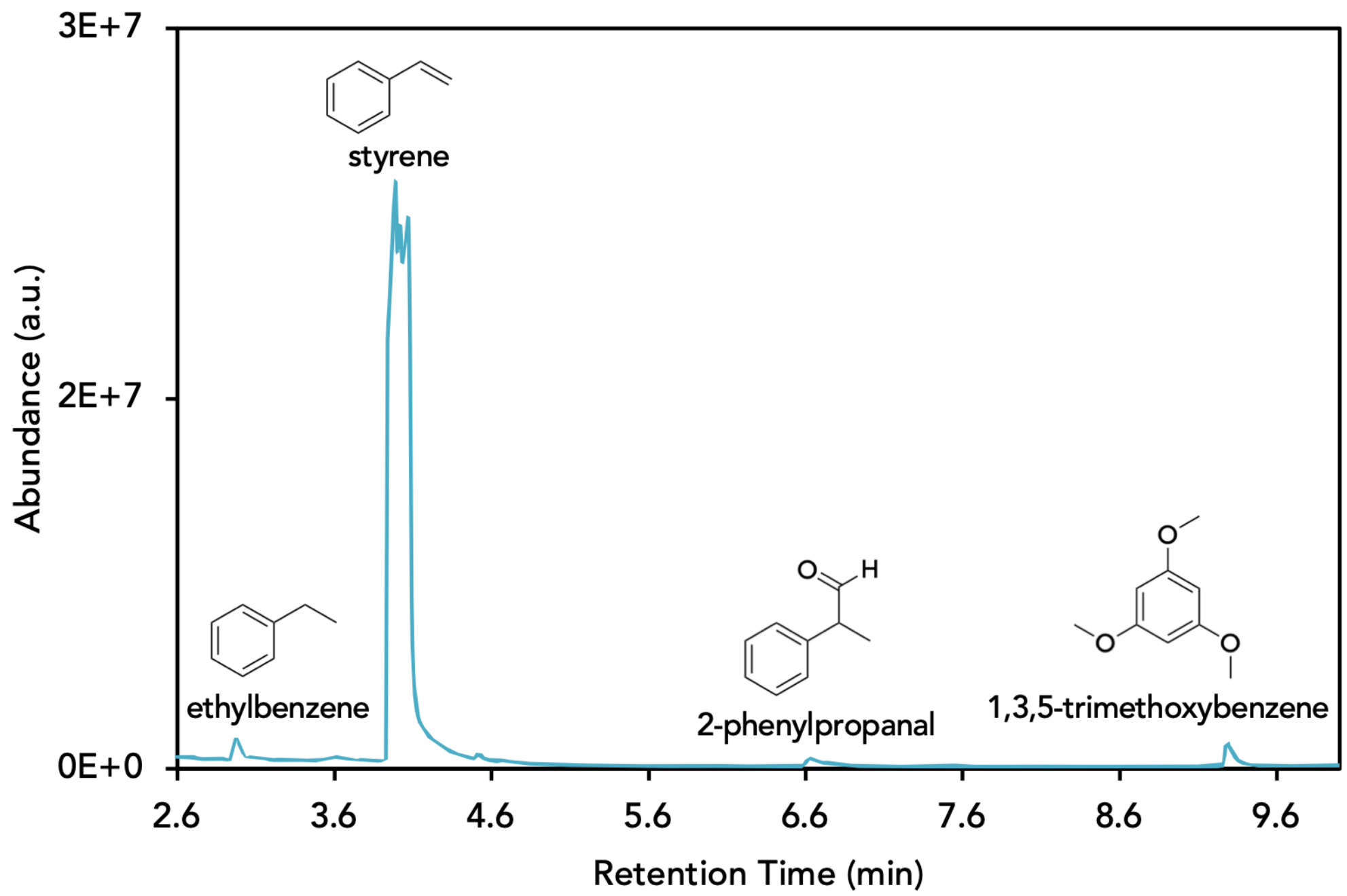

Figure S2. Example gas chromatogram of ethylbenzene, styrene, 2-phenylpropanal, and 1,3,5-trimethoxybenzene.

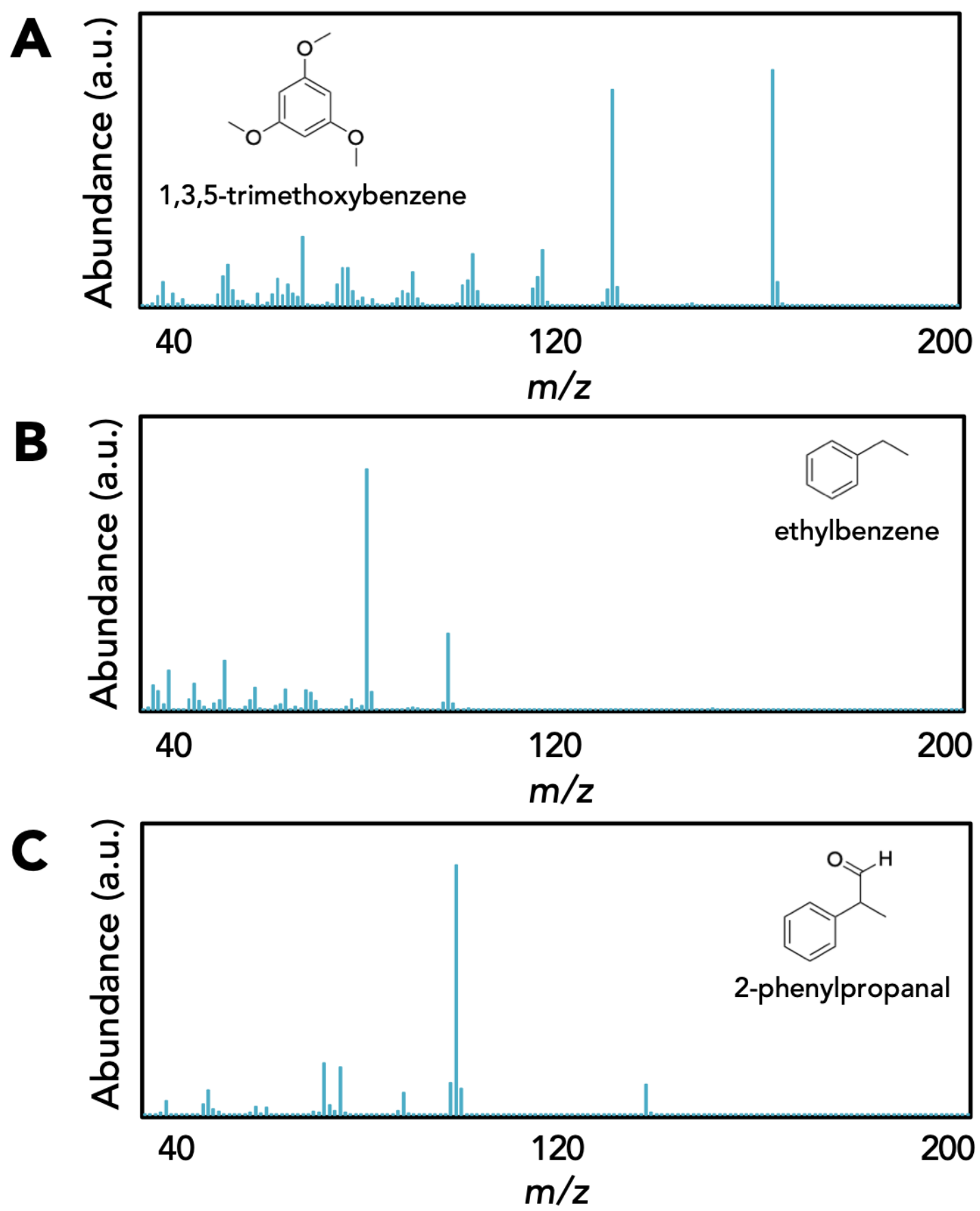

Figure S3. Mass spectra of **A**) 1,3,5-trimethoxybenzene, **B**) ethylbenzene, and **C**) 2-phenylpropanal.

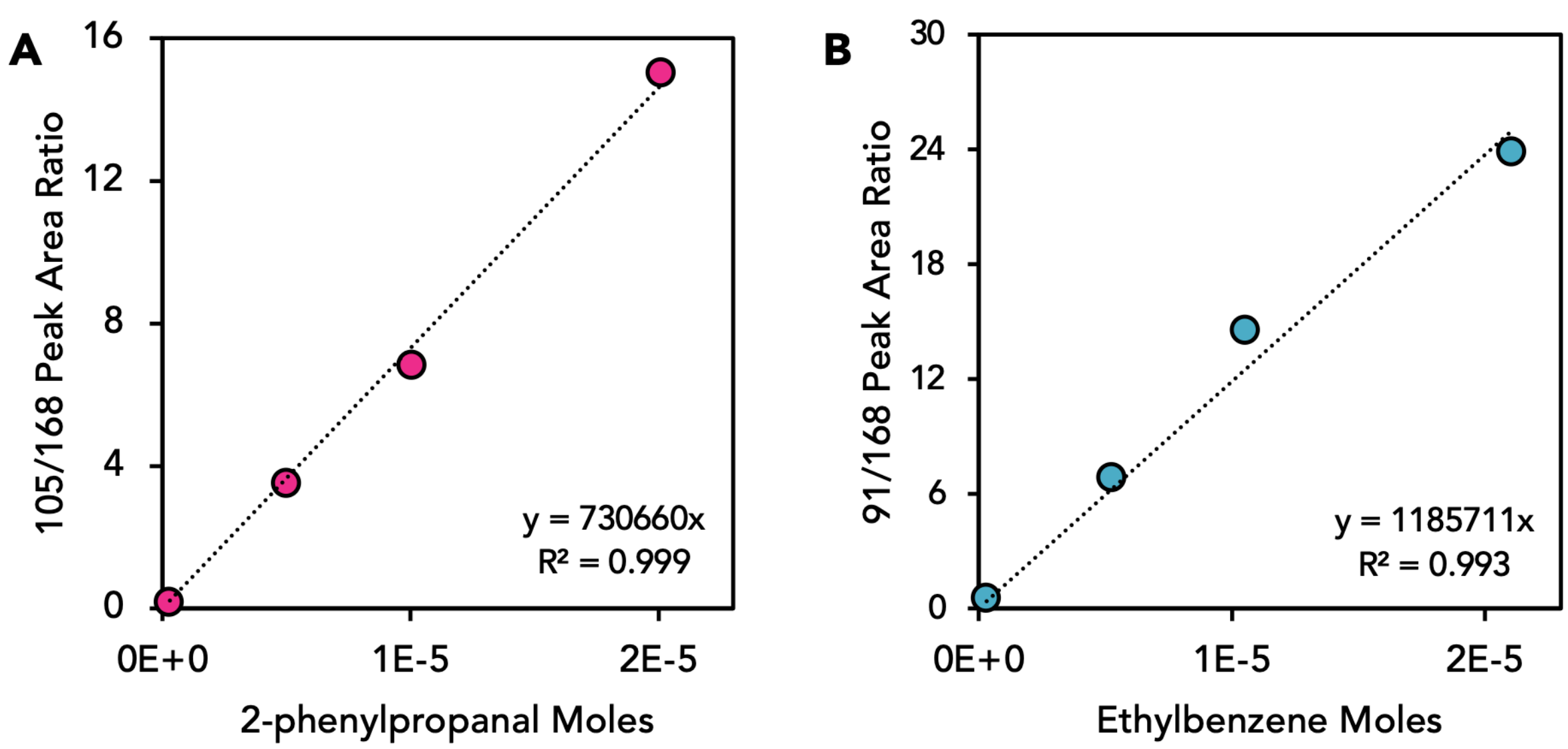

Figure S4. Standard curves of **A**) 2-phenylpropanal and **B**) ethylbenzene using TMB as an internal standard.

Equation S1. FE calculation for GC-MS and NMR quantification

$$FE\ (\%) = \frac{2\left(\frac{\text{mol e}^-}{\text{mol product}}\right) * \text{Product amount (mol product)} * F\left(\frac{\text{C}}{\text{mol e}^-}\right)}{\text{Total charge passed (C)}} * 100$$

Equation S2. Partial current density calculation for GC-MS and NMR quantification

$$j_{\text{electro-HFN}}\ (\text{mA cm}^{-2}) = \frac{\text{Total charge passed (C)} * \frac{FE\ (\%)}{100} * 1000\ (\frac{\text{mA}}{\text{A}})}{\text{Time (s)} * \text{Area}_{\text{geometric}}\ (\text{cm}^2)}$$

1.8 Product Quantification via Nuclear Magnetic Resonance Spectroscopy

To quantify 2-phenylpropanal, we ran ^{1}H NMR spectroscopy experiments on a Bruker 400 MHz spectrometer (**Figure S5**). After electrolysis, 200 µL of an aqueous 0.4 M formic acid (95%, Sigma-Aldrich) solution was added to the electrolyte while the electrolyte was stirring. Then, the electrolyte was pipetted into a 20 mL scintillation vial. The cell was rinsed with 700 µL of acetone and the rinse was added to the 20 mL scintillation vial. Notably, the electrolyte became a single phase upon addition of acetone. For solvent and supporting electrolyte salt screening, the volume of acetone was increased to 1.4 mL to ensure that a single-phase solution was achieved. Finally, NMR samples were prepared by adding 200 µL of the single-phase electrolyte solution to 400 µL of dimethyl sulfoxide-d_6 (99.9%, Cambridge Isotope Laboratories). The FE and partial current density were calculated using the moles of product detected by NMR and the geometric surface area (1 cm^2) (**Equations S1 and S2**).

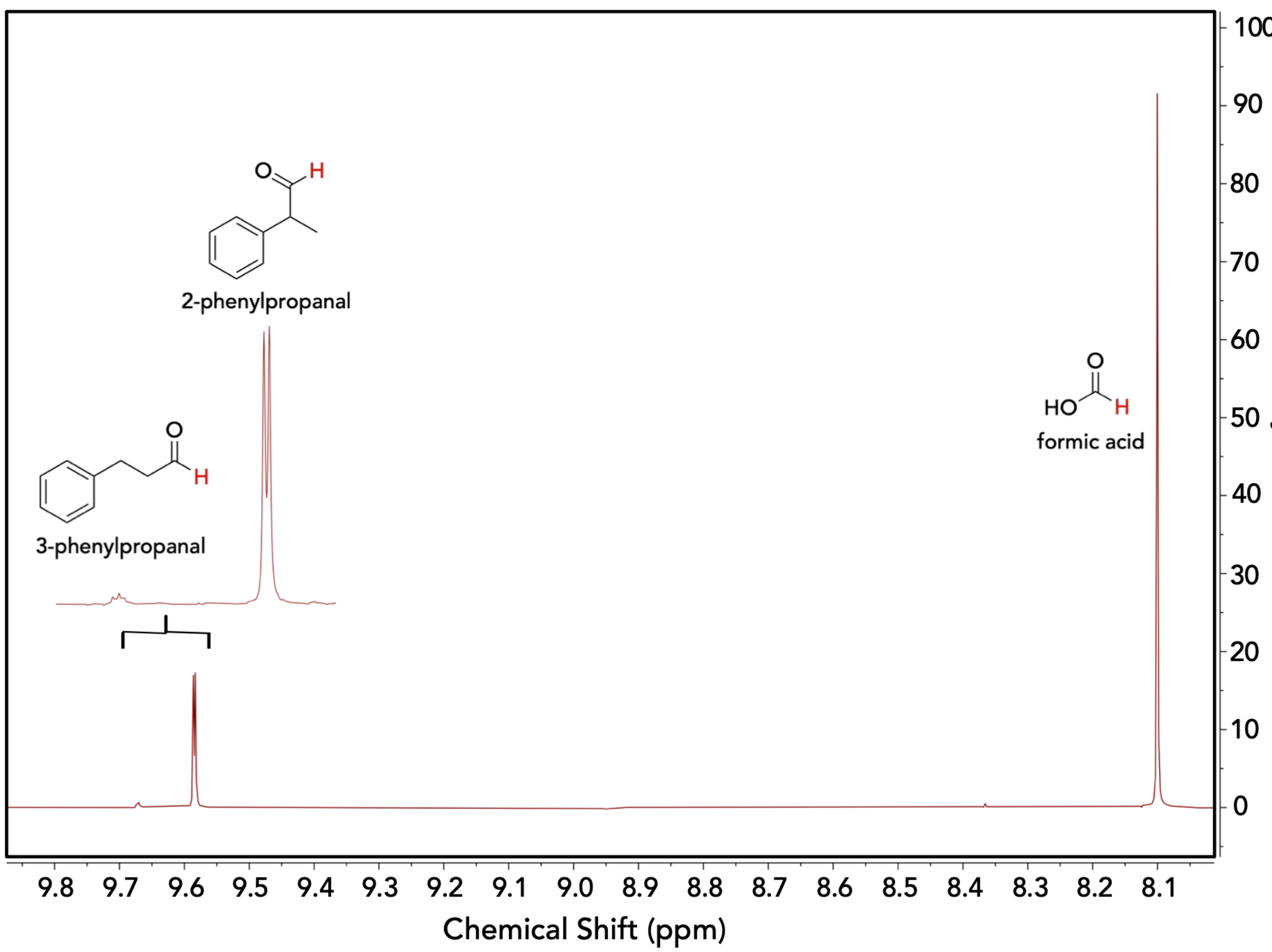

Figure S5. Example ^{1}H NMR spectra of the aldehydic peaks of 3-phenylpropanal, 2-phenylpropanal, and formic acid in dimethyl sulfoxide-d_6 at 9.67, 9.58, and 8.10 ppm, respectively.

1.9 H_2 Quantification via Gas Chromatography with a Thermal Conductivity Detector

To quantify H_2, we used an SRI 8610C GC-TCD instrument fitted with a molecular-sieve column (**Figure S6**). Argon was used as the carrier gas. First, we generated a standard curve of known H_2 concentrations by changing the relative flow rates of the H_2 and N_2 streams while maintaining a total flow rate of 60 sccm through the 1 mL sample loop (**Figure S7**). The temperature sequence was programmed to begin at 40 °C for 2 minutes, then ramp to 150 °C at 50 °C min^{-1} and hold at 150 °C for 4 minutes.

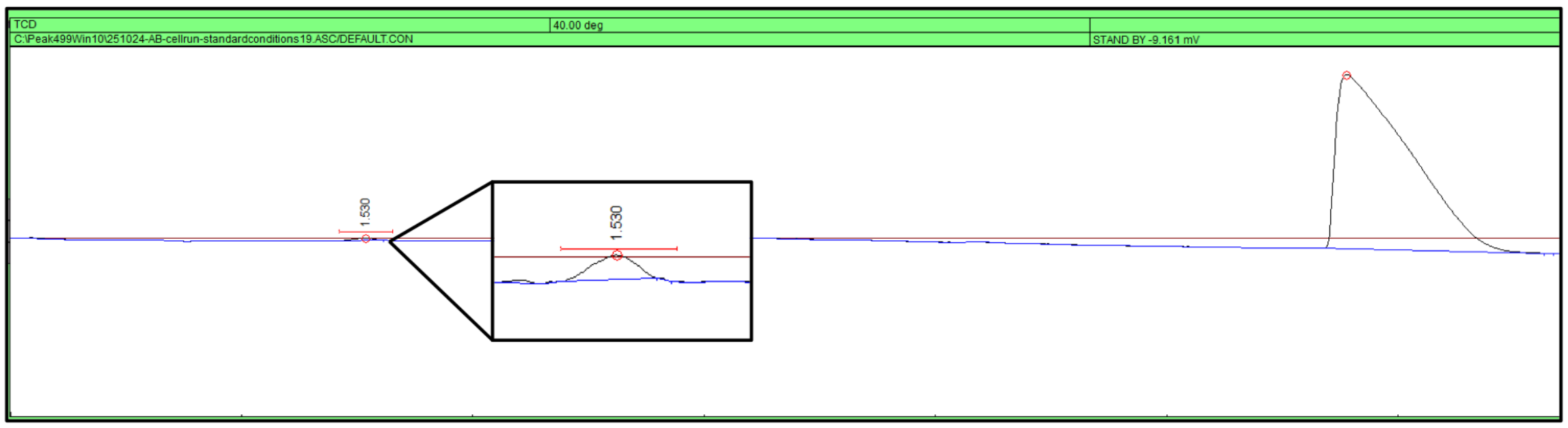

Figure S6. Example TCD chromatogram of H_2 and CO detected at 1.5 and 5.8 minutes, respectively.

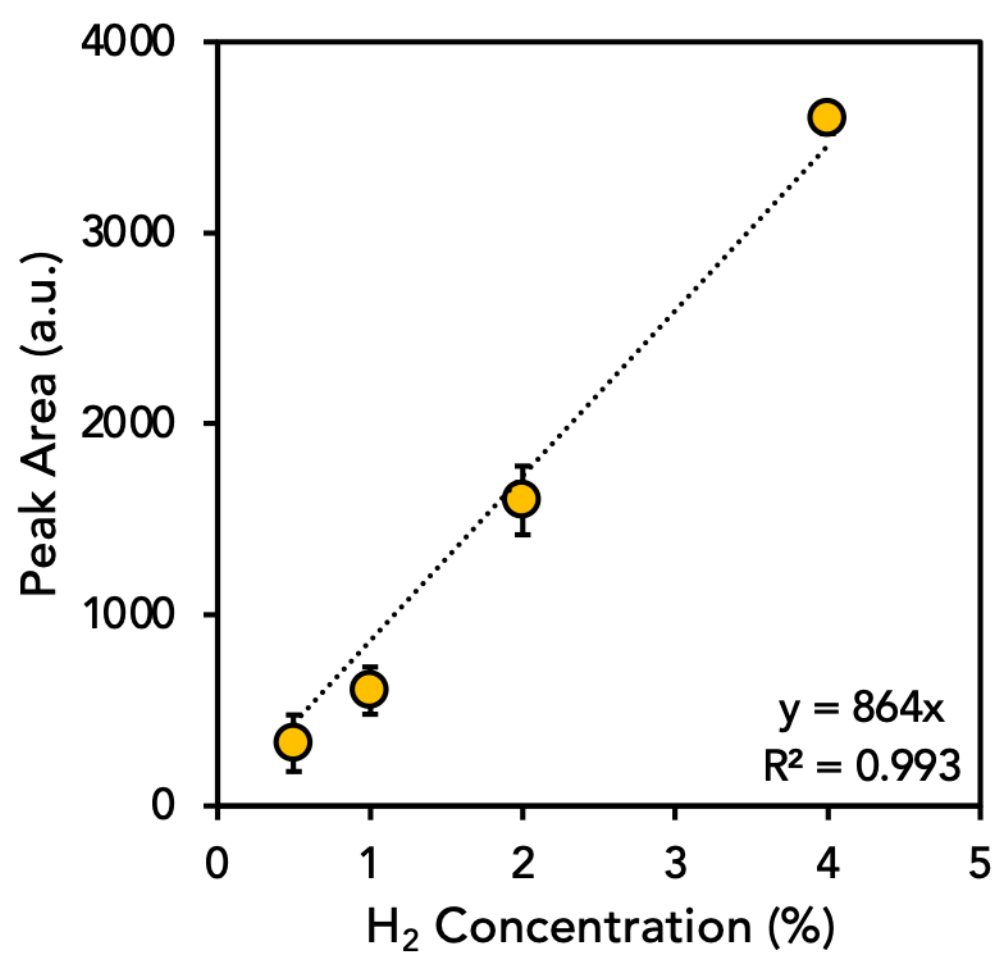

Figure S7. Standard curve of H_2 concentrations via GC-TCD. Error bars represent one standard deviation with $n \geq 2$.

To quantify H_2 during an electrolysis, a back pressure regulator (Zaiput BPR-10) was plumbed directly downstream of the electrochemical cell to maintain a pressure of 5 bar CO in the reactor headspace during flow. To achieve a flow rate of 3 sccm through the reactor and into the sample loop, the BPR pressure was set to 4.9 bar. To increase the detected peak area of H_2 at low potentials, the flow rate was lowered to 1 and 2 sccm at –0.66 and –0.76 V vs. SHE, respectively.

After the typical pressurization procedure detailed in Section 1.6 was complete, the MFC was set to the desired flow rate (1–3 sccm). For a typical 30-minute electrolysis, the GC-TCD sample sequence was initiated at 15 and 30 minutes into the electrolysis. The H_2 peak areas were averaged between these time points. The FE of H_2 was calculated using the total flow rate, % H_2, total charge passed, and length of the electrolysis (**Equation S3**).

Equation S3. FE calculation for GC-TCD

$$FE(\%) = 100 * \frac{\text{Flow rate}\left(\frac{\text{mL}}{\text{s}}\right) * \left(\frac{\%\ \text{H}_2}{100}\right) * \left(\frac{1}{22400}\right)\left(\frac{\text{mol H}_2}{\text{mL H}_2}\right) * 2\left(\frac{\text{mol e}^-}{\text{mol H}_2}\right) * F\left(\frac{\text{C}}{\text{mol e}^-}\right)}{\frac{\text{Charge passed (C)}}{\text{Length of electrolysis (s)}}}$$

1.10 Powder X-ray Diffraction

Powder XRD experiments were performed using a Rigaku SmartLab diffractometer (Cu Kα). XRD scans were collected from 5° to 65° using a 10° min^{-1} scan rate. Samples were prepared by pressurization of the standard electrolyte at 1 and 5 bar CO for 30 minutes, followed by centrifugation at 19,090 rcf for 5 minutes. The supernatant was decanted and the precipitate was air-dried overnight.

1.11 Attenuated Total Reflectance Fourier Transform Infrared Absorption Spectroscopy

Ex situ ATR-FTIR spectroscopy measurements were performed using a Nicolet iS50 Fourier transform infrared spectrometer. To measure the sample at 1 bar CO, CO was bubbled through the electrolyte in the electrochemical cell for 30 minutes while stirring. To measure the sample at 5 bar CO, CO was bubbled through the electrolyte in the electrochemical cell for 5 minutes and then the cell was pressurized to 5 bar CO and left for 30 minutes. Then, the cell was depressurized and 1 mL of the sample was centrifuged at 19,090 rcf for 5 minutes to achieve complete phase separation and removal of the $Rh_6(CO)_{16}$ precipitate. Finally, 40 µL of the organic phase was dropped onto a silicon crystal for measurement. The background for each sample was an identically prepared electrolyte without Rh precatalyst that had undergone the same CO and centrifugation treatment.

1.12 UV-visible Spectroscopy

Ex situ UV-Vis spectroscopy measurements were performed using an Agilent Cary 60 UV-Vis spectrophotometer. A reference spectrum was collected on a sample of the standard electrolyte without the precatalyst and subtracted from the sample spectrum. To measure the sample at 1 bar CO, CO was bubbled through the electrolyte in the electrochemical cell for 30 minutes. To measure the sample at 5 bar CO, CO was bubbled through the electrolyte in the electrochemical cell for 5 minutes and then the cell was pressurized to 5 bar CO for 30 minutes. Then, the cell was depressurized and 1 mL of the sample was centrifuged at 19,090 rcf for 5 minutes to achieve complete phase separation and removal of the $Rh_6(CO)_{16}$ precipitate. Finally, 20 µL of the organic phase was diluted 250-fold with IPA and measured in a quartz cuvette.

1.13 X-ray Photoelectron Spectroscopy

X-ray photoelectron spectroscopy (XPS) data were collected using a Kratos AXIS Ultra spectrometer. Samples were irradiated with a monochromatic Al Kα x-ray source (1486.6 eV) at 150 W at pressures $< 1 \times 10^{-9}$ Torr. The analyzer pass energy was set to 80 eV for survey scans and 10 eV for high-resolution scans, and the neutralizer was used to mitigate charge buildup on insulating samples. XPS spectra were analyzed using CasaXPS software and calibrated to adventitious C at 284.8 eV. A Shirley-type background was subtracted from all spectra prior to peak fitting. Different line shapes of LA(50) and GS(30) were used for Rh(0) and Rh(III) components, respectively. The areas of Rh $3d_{5/2}$ peaks were constrained by their Rh $3d_{3/2}$ counterparts with a 3:2 $3d_{5/2}$ to $3d_{3/2}$ area ratio. Additionally, Rh 3d peak positions were constrained by a spin–orbit splitting value of ΔE(Rh 3d) = 4.71 eV.

1.14 Scanning Electron Microscopy and Energy-Dispersive X-ray Spectroscopy

Scanning electron microscopy (SEM) images and energy-dispersive X-ray spectroscopy (EDS) elemental maps were collected using a ZEISS 1550VP high-resolution analytical scanning electron microscope equipped with an Oxford X-Max silicon drift EDS detector. After electrolysis, cathodes were rinsed with acetone and dried in air, then mounted on an aluminum stub with conductive carbon tape. No conductive coating was applied. Images were collected using an in-lens secondary electron detector at an accelerating voltage of 10 kV and a working distance of 7.8 mm. EDS maps were acquired at an accelerating voltage of 15 kV and processed using AZtec.

2. Supplemental Data and Discussions

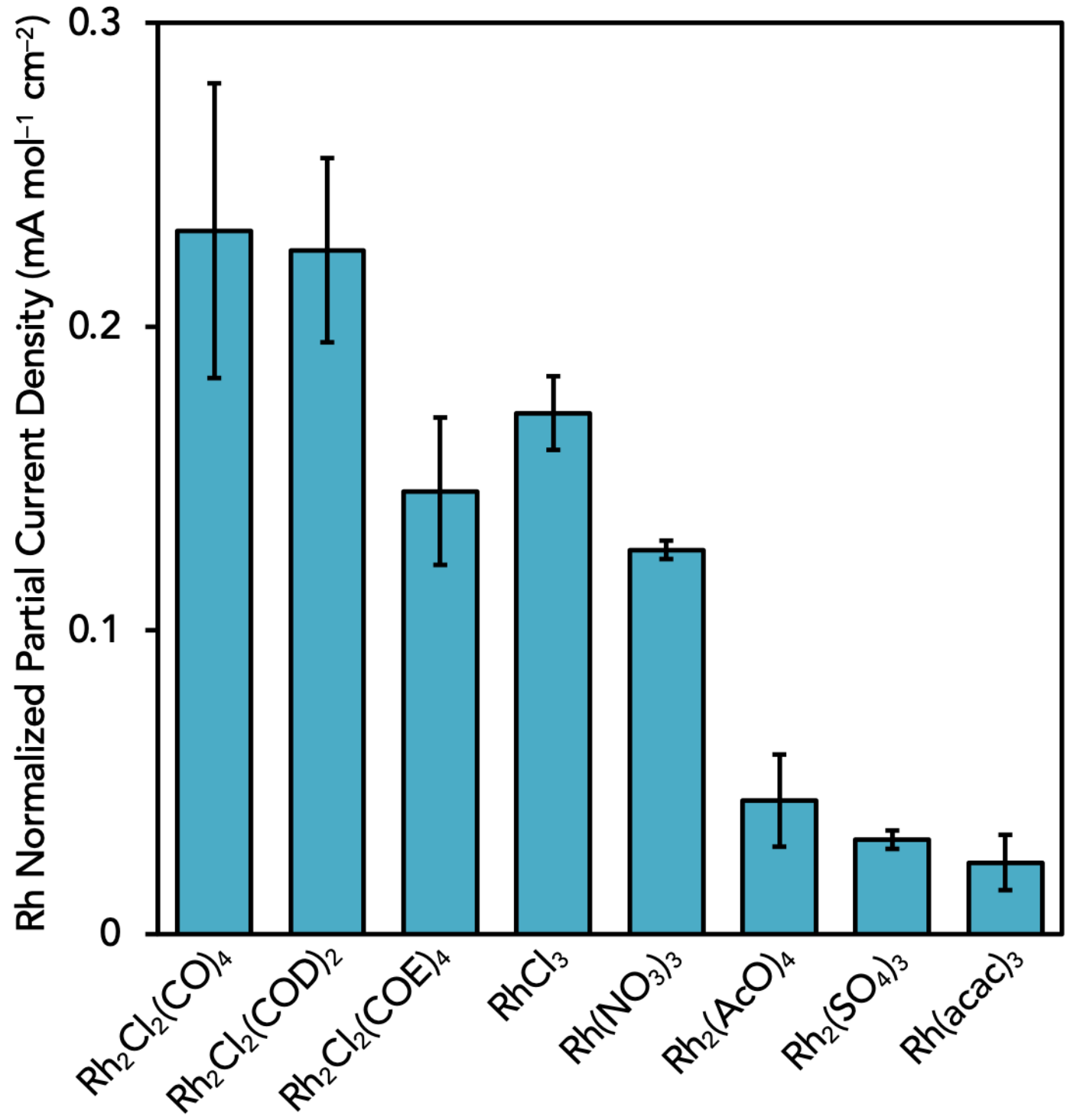

Figure S8. Partial current density of Rh complexes normalized by Rh moles. Error bars represent one standard deviation with $n \geq 2$. Experimental conditions unless stated otherwise: 3 mM $Rh_2Cl_2(CO)_4$, 0.52 M styrene, 0.1 M TBAOTf, 50/50 v/v IPA/H_2O, Toray 120 (5% Teflon) carbon paper cathode, 5 bar CO, –0.91 V vs. SHE.

Supplemental Discussion 1. Explanation of Rh plating behavior among complexes

As described in Section 2.1 in the main text, at 0.52 M styrene, Rh complexes either partitioned into the styrene phase or aqueous phase. $Rh_2Cl_2(CO)_4$, $Rh_2Cl_2(COE)_4$, $Rh_2Cl_2(COD)_2$, and $Rh(acac)_3$ were soluble in the styrene phase, while $RhCl_3{\cdot}xH_2O$, $Rh(NO_3)_3{\cdot}xH_2O$, $Rh_2(OAc)_4$, and $Rh_2(SO_4)_3$ were soluble in the aqueous phase. At 0.52 M styrene, black deposits were visually observed on the cathode when the electrolytes contained $RhCl_3{\cdot}xH_2O$, $Rh(NO_3)_3{\cdot}xH_2O$, and $Rh_2(SO_4)_3$. However, at 0.28 M styrene, when the electrolyte was a single aqueous phase, black deposits were observed for all Rh complexes except $Rh_2(OAc)_4$. Despite $Rh_2Cl_2(CO)_4$, $Rh_2Cl_2(COE)_4$, $Rh_2Cl_2(COD)_2$, and $Rh(acac)_3$ resisting Rh deposit formation at 0.52 M styrene, at sufficiently low CO pressures (< 2 bar), these complexes produced deposits as well.

We propose that this could be explained by the ability of the aqueous phase to better facilitate anionic ligand dissociation due to its higher ionic conductivity (0.1 M TBAOTf), compared to the nonpolar styrene phase. Ligand dissociation is a necessary step to reduce a solvated Rh cation to solid Rh(0). Additionally, this could also be the result of differences in electrode wetting between the styrene phase and aqueous phase, which dictates the amount of Rh that is electrochemically accessible to the cathode.

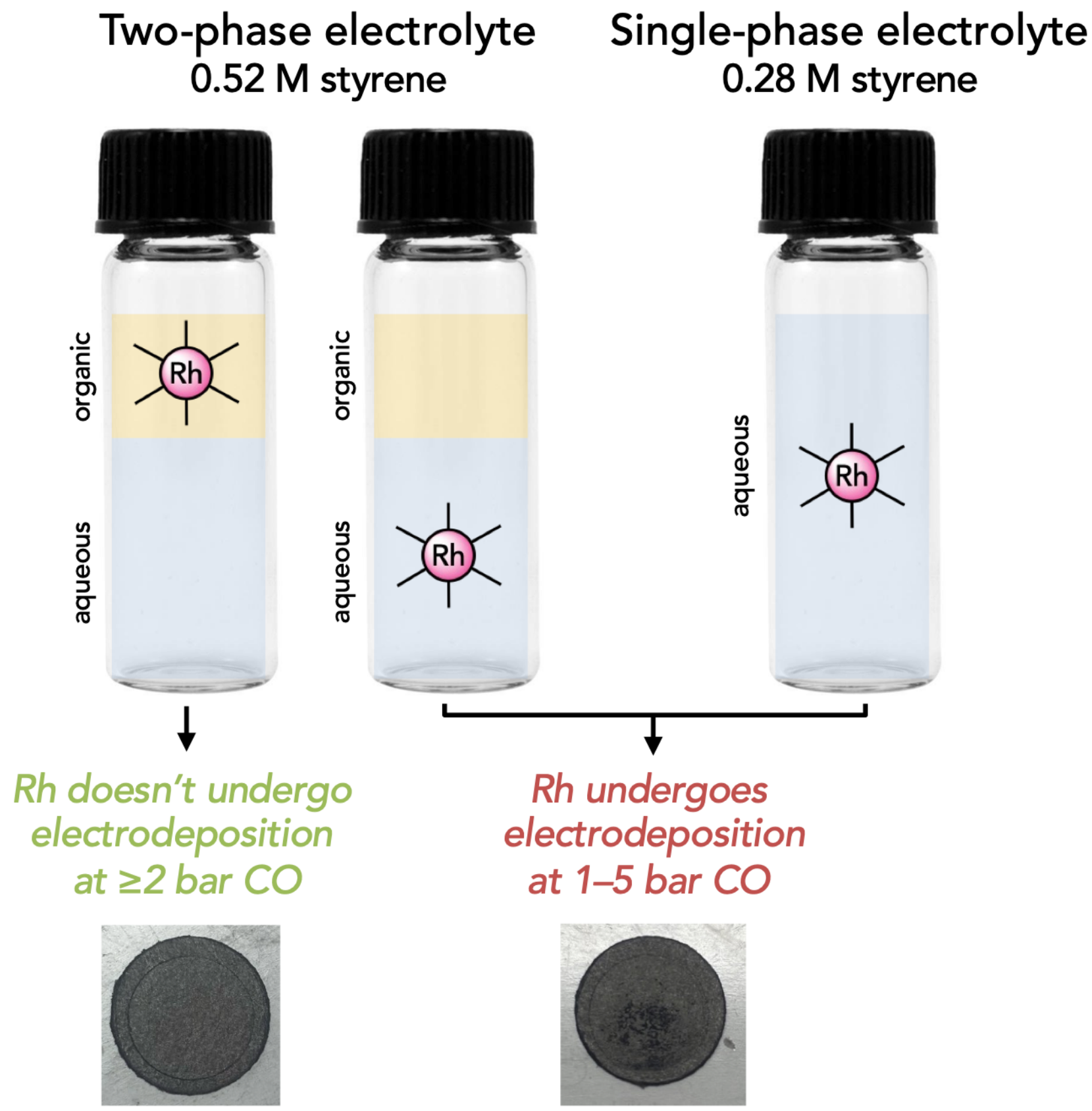

Figure S9. Schematic of the conditions (electrolyte and CO) that result in the formation of Rh deposits and corresponding images of carbon cathodes with and without Rh deposits.

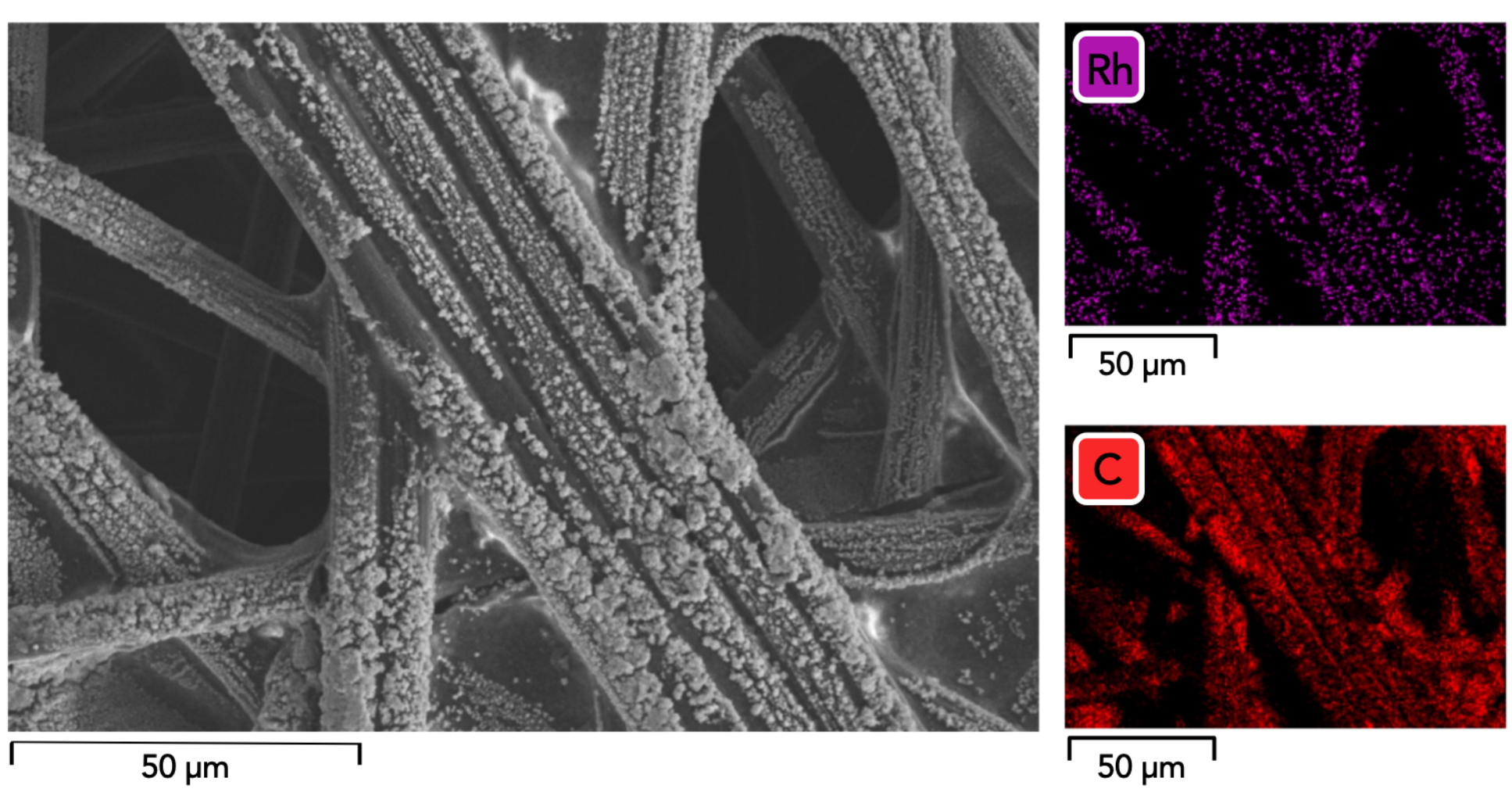

Figure S10. Evidence of Rh-containing deposits on the cathode after electrolysis with $RhCl_3 \cdot xH_2O$ at 5 bar CO. Scanning electron microscopy of the cathode after electrolysis and energy-dispersive X-ray spectroscopy elemental mapping. Experimental conditions: 3 mM $RhCl_3$, 0.52 M styrene, 0.1 M TBAOTf, 50/50 v/v IPA/H_2O, Toray 120 (5% Teflon) carbon paper cathode, 5 bar CO, –1.11 V vs. SHE.

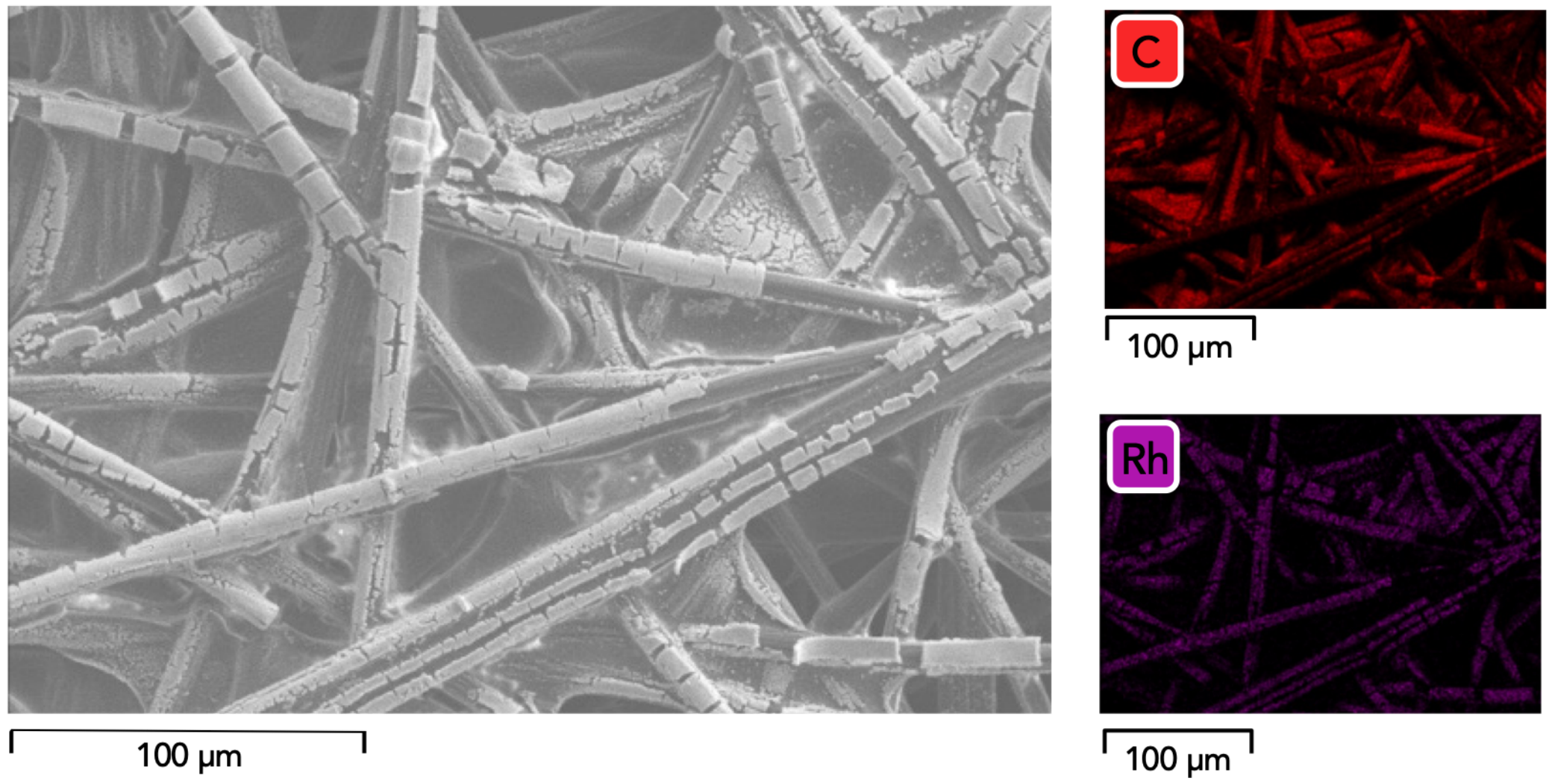

Figure S11. Evidence of Rh-containing deposits on the cathode after electrolysis with $Rh_2Cl_2(CO)_4$ at 1 bar CO. Scanning electron microscopy of the cathode after electrolysis and energy-dispersive X-ray spectroscopy elemental mapping. Experimental conditions: 3 mM $Rh_2Cl_2(CO)_4$, 0.52 M styrene, 0.1 M TBAOTf, 50/50 v/v IPA/H_2O, Toray 120 (5% Teflon) carbon paper cathode, 1 bar CO, –1.11 V vs. SHE.

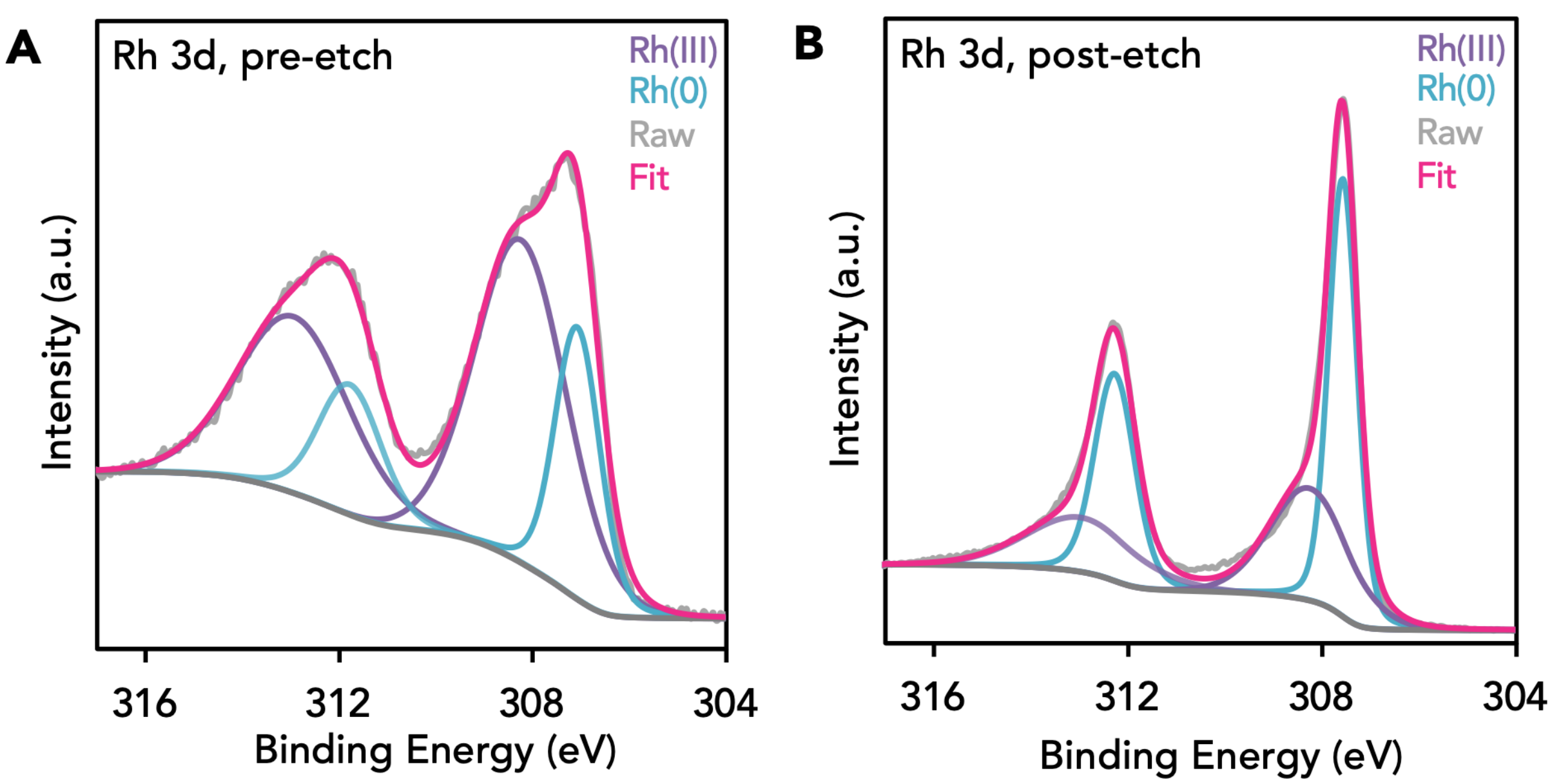

Figure S12. Rh 3d X-ray photoelectron spectroscopy of a carbon paper cathode with Rh deposits after electrolysis. **A**) Before Ar etching and **B**) after Ar etching. Experimental conditions: 3 mM $Rh_2Cl_2(CO)_4$, 0.52 M styrene, 0.1 M TBAOTf, 50/50 v/v IPA/H_2O, Toray 120 (5% Teflon) carbon paper cathode, 1 bar CO, –1.11 V vs. SHE.

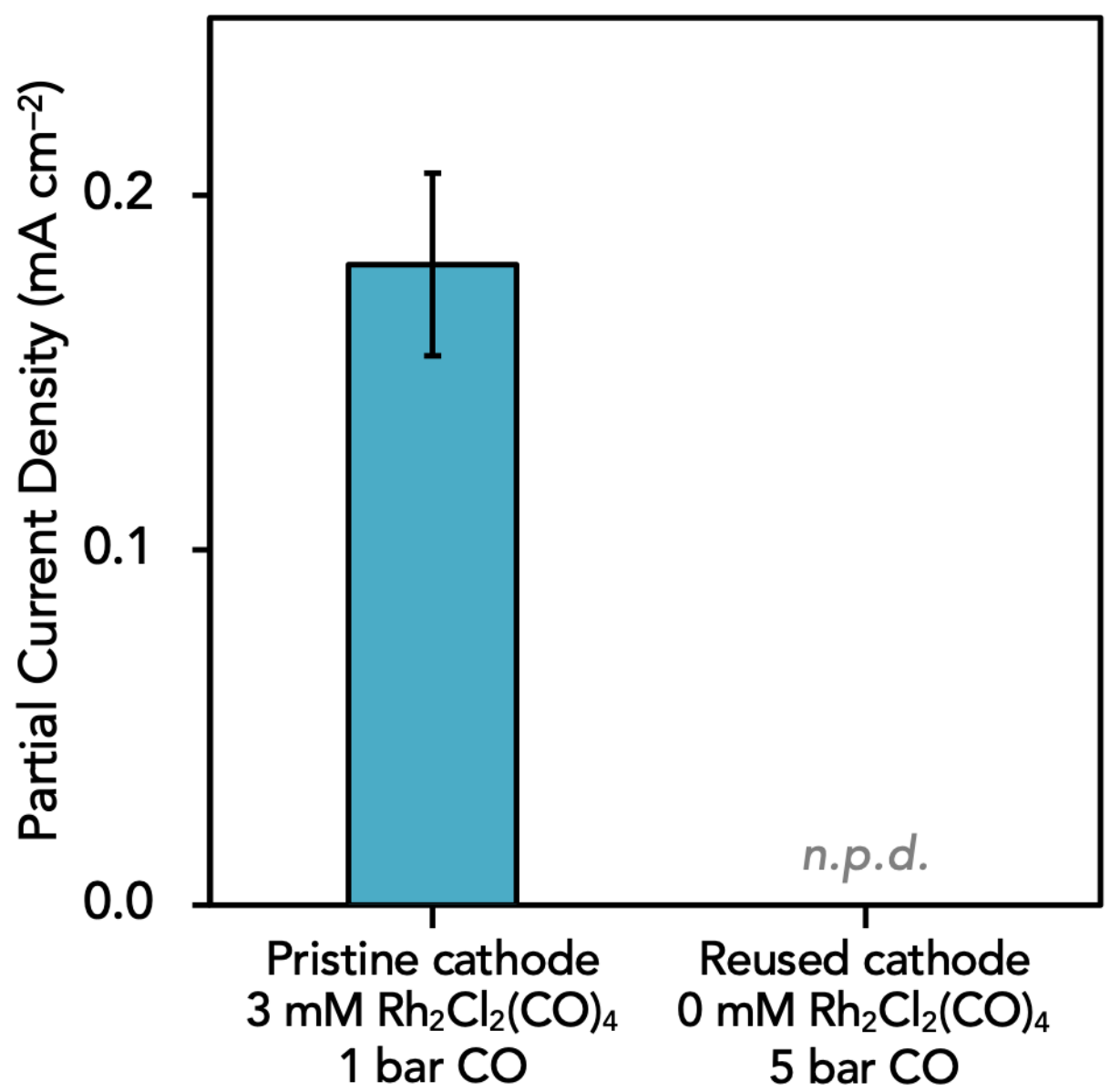

Figure S13. Control experiments excluding the possibility of Rh-containing deposits contributing to product formation. Electrolysis using a pristine carbon paper cathode and 3 mM $Rh_2Cl_2(CO)_4$ at 1 bar CO (**left**) and the reused carbon paper from the previous experiment with fresh electrolyte containing no $Rh_2Cl_2(CO)_4$ at 5 bar CO (**right**); n.p.d. = no product detected. Error bars represent one standard deviation with $n \geq 2$. Experimental conditions unless stated otherwise: 3 mM $Rh_2Cl_2(CO)_4$, 0.52 M styrene, 0.1 M TBAOTf, 50/50 v/v IPA/H_2O, Toray 120 (5% Teflon) carbon paper cathode, –1.11 V vs. SHE.

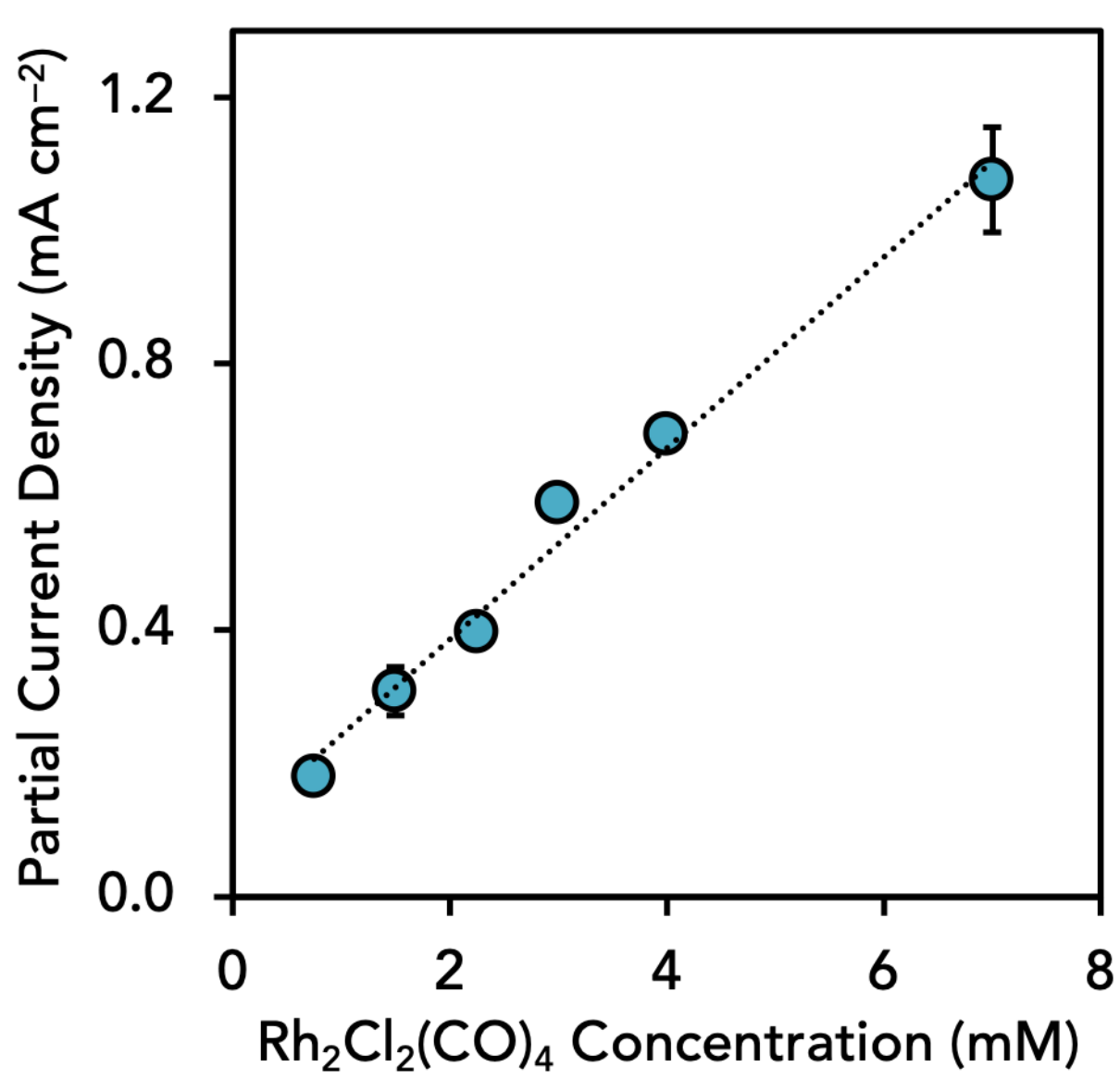

Figure S14. $Rh_2Cl_2(CO)_4$ concentration dependence. Error bars represent one standard deviation with $n \geq 2$. Experimental conditions: 0.52 M styrene, 0.1 M TBAOTf, 50/50 v/v IPA/H_2O, Toray 120 (5% Teflon) carbon paper cathode, 5 bar CO, –1.11 V vs. SHE.

Supplemental Discussion 2. Capacitance measurements as cathode ECSA estimates

To account for the effect of differences in electrode surface area on the partial current, we calculated the apparent electrochemically active surface area (ECSA). First, we ran cyclic voltammograms of the standard electrolyte (0.52 M styrene and 0.1 M TBAOTf in 50/50 v/v IPA/H_2O) at 5 bar CO using the pressurization procedure described in Section 1.6. The voltammograms were run ± 50 mV from open circuit potential (OCP) at four different scan rates (ν), 10, 50, 150, and 200 mV s^{-1}, and the average absolute values of the anodic and cathodic capacitive currents (i_{avg}) were recorded. C_{dl} was calculated as the slope of i_{avg} vs. ν (**Equation S4**). Then, apparent ECSA was calculated using C_{dl} and the specific capacitance (C_{s}) of an atomically smooth planar surface of a material at a given electrolyte condition (**Equation S5**).

Obtaining accurate C_{s} values is challenging because most electrode–electrolyte compositions do not have reported C_{s} values in the literature and synthesizing the corresponding materials and measuring these values experimentally is not always practical. However, we estimated ECSA using a standard C_{s} value for metal foils (20 μF cm^{-2})[3] and we saw that the trend remained relatively the same with the exception of glassy carbon possessing the highest partial current density (**Figure S15, ECSA Normalization 1**). Additionally, we estimated ECSA using reported C_{s} values specific to our cathode materials, which correct for differences between carbonaceous and oxide-forming cathode materials.[4] Using this method, glassy carbon still possessed the highest partial current density, followed by carbon paper (**Figure S15, ECSA Normalization 2**). It is important to note that these literature C_{s} values are approximate and do not reflect the differences that arise due to the particular electrolyte we used in this study. Overall, these data indicated that glassy carbon electrodes possess the highest partial current density when normalized by ECSA using approximate C_{s} values. However, because of the uncertainty on C_{s} and ECSA values, we chose to normalize the partial current densities in Figure 2B by the geometric surface area. Therefore, CP was chosen as the cathode for subsequent experiments because it produced the highest partial current density normalized to geometric surface area.

Equation S4. Double layer capacitance

$$C_{\mathrm{dl}} = \frac{\mathrm{d}i_{\mathrm{avg}}}{\mathrm{d}\nu}$$

Equation S5. Electrochemically active surface area

$$\mathrm{ECSA} = \frac{C_{\mathrm{dl}}}{C_{\mathrm{s}}}$$

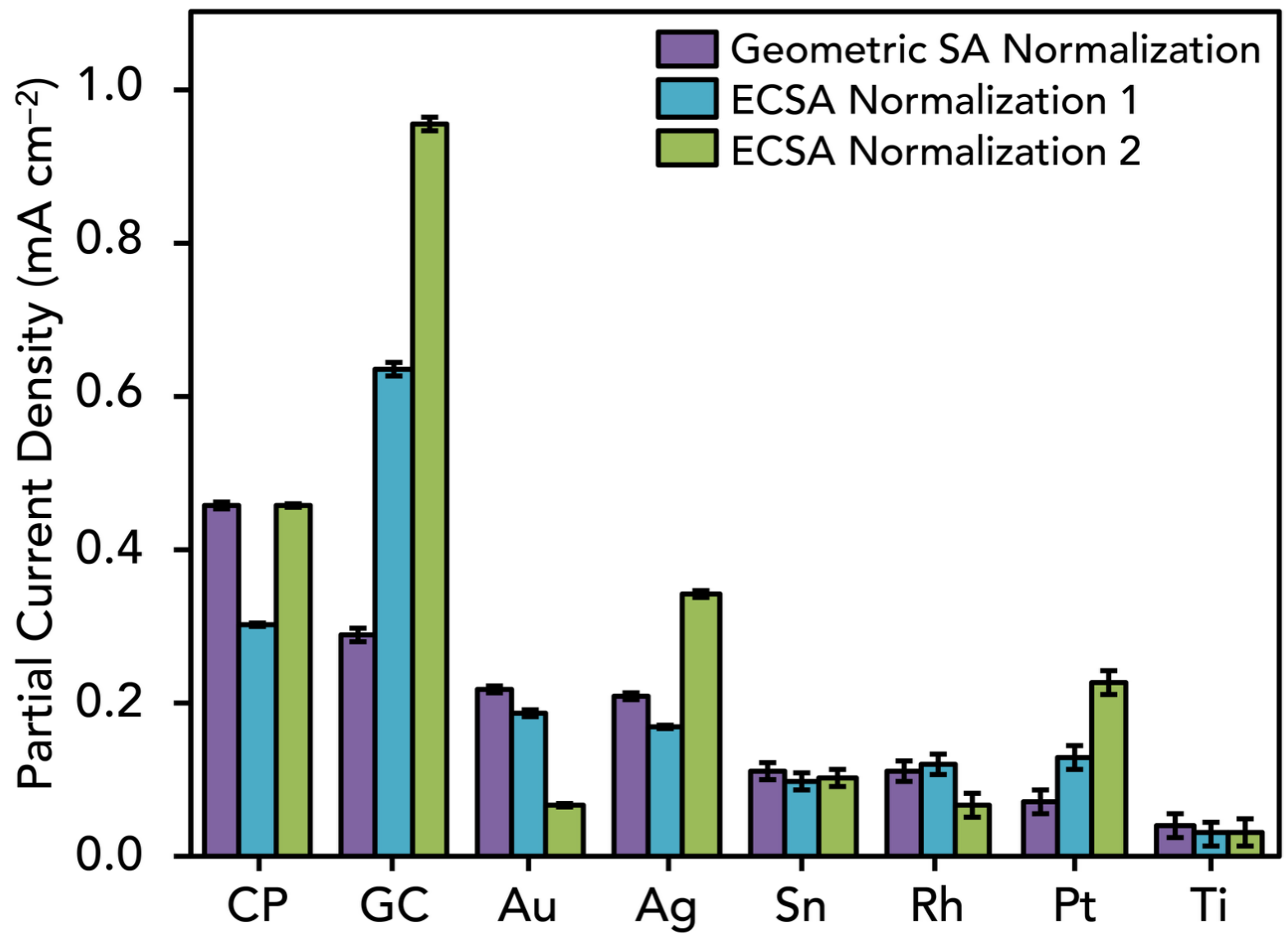

Figure S15. Partial current normalization by geometric surface area (1 cm^2) and electrochemically active surface area (ECSA) for cathode materials. ECSA Normalization 1 uses C_s = 20 µF cm^{-2} for all electrodes. ECSA Normalization 2 uses different C_s values for each electrode based on literature reports. Error bars represent one standard deviation with n ≥ 2. Experimental conditions: 3 mM $Rh_2Cl_2(CO)_4$, 0.52 M styrene, 0.1 M TBAOTf, 50/50 v/v IPA/H_2O, 5 bar CO, –0.91 V vs. SHE.

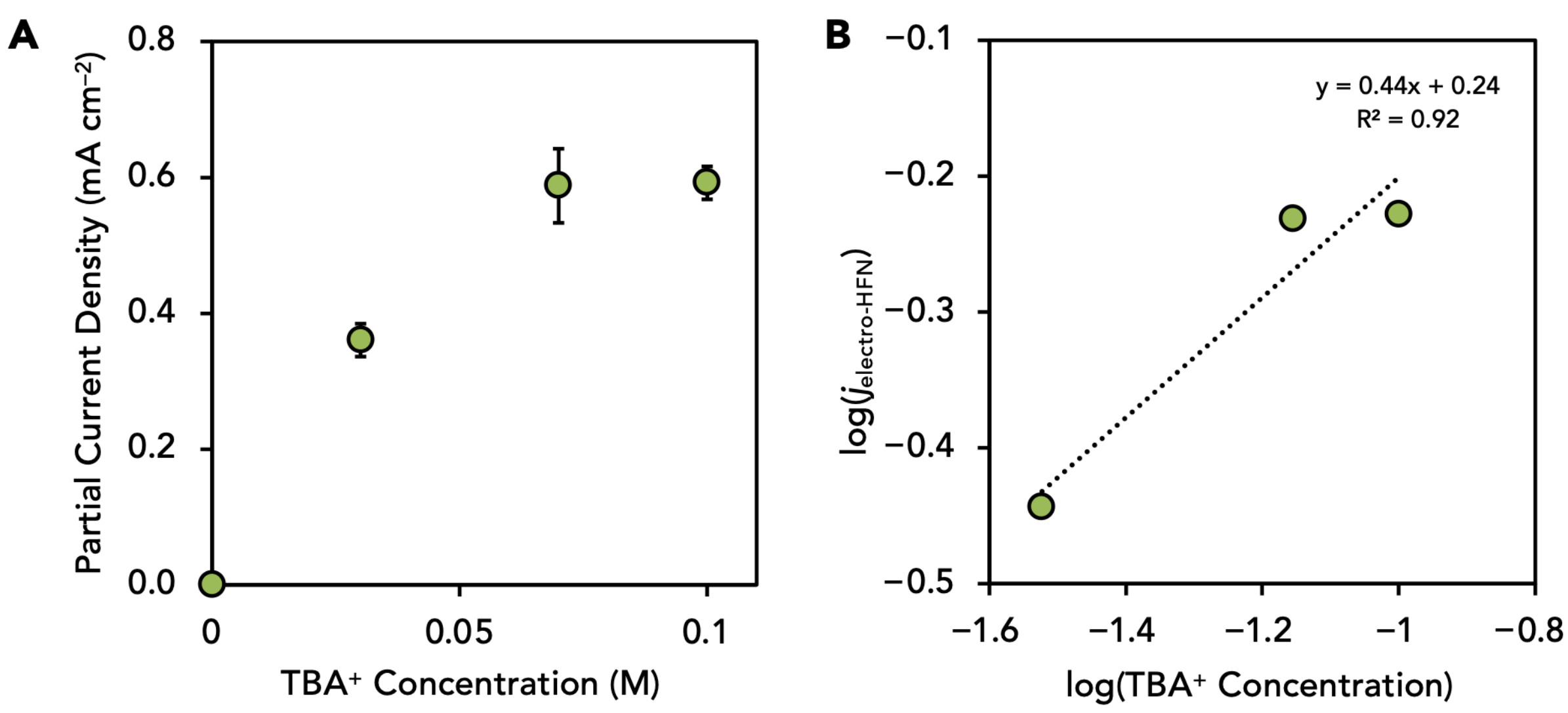

Figure S16. A) Tetrabutylammonium (TBA^+) concentration dependence. **B**) TBA^+ concentration dependence on a log–log plot. We computed an apparent order of 0.44 (R^2 = 0.92), but we do not believe this represents a kinetic rate order due to the many roles of TBA^+ discussed in Section 2.1 in the main text. KOTf was added to maintain an ionic strength of 0.1 M (e.g., 0.07 M TBAOTf and 0.03 M KOTf). Error bars represent one standard deviation with n ≥ 2. Experimental conditions unless stated otherwise: 3 mM $Rh_2Cl_2(CO)_4$, 0.52 M styrene, 0.1 M supporting electrolyte salt, 50/50 v/v IPA/H_2O, Toray 120 (5% Teflon) carbon paper cathode, 5 bar CO, –1.11 V vs. SHE.

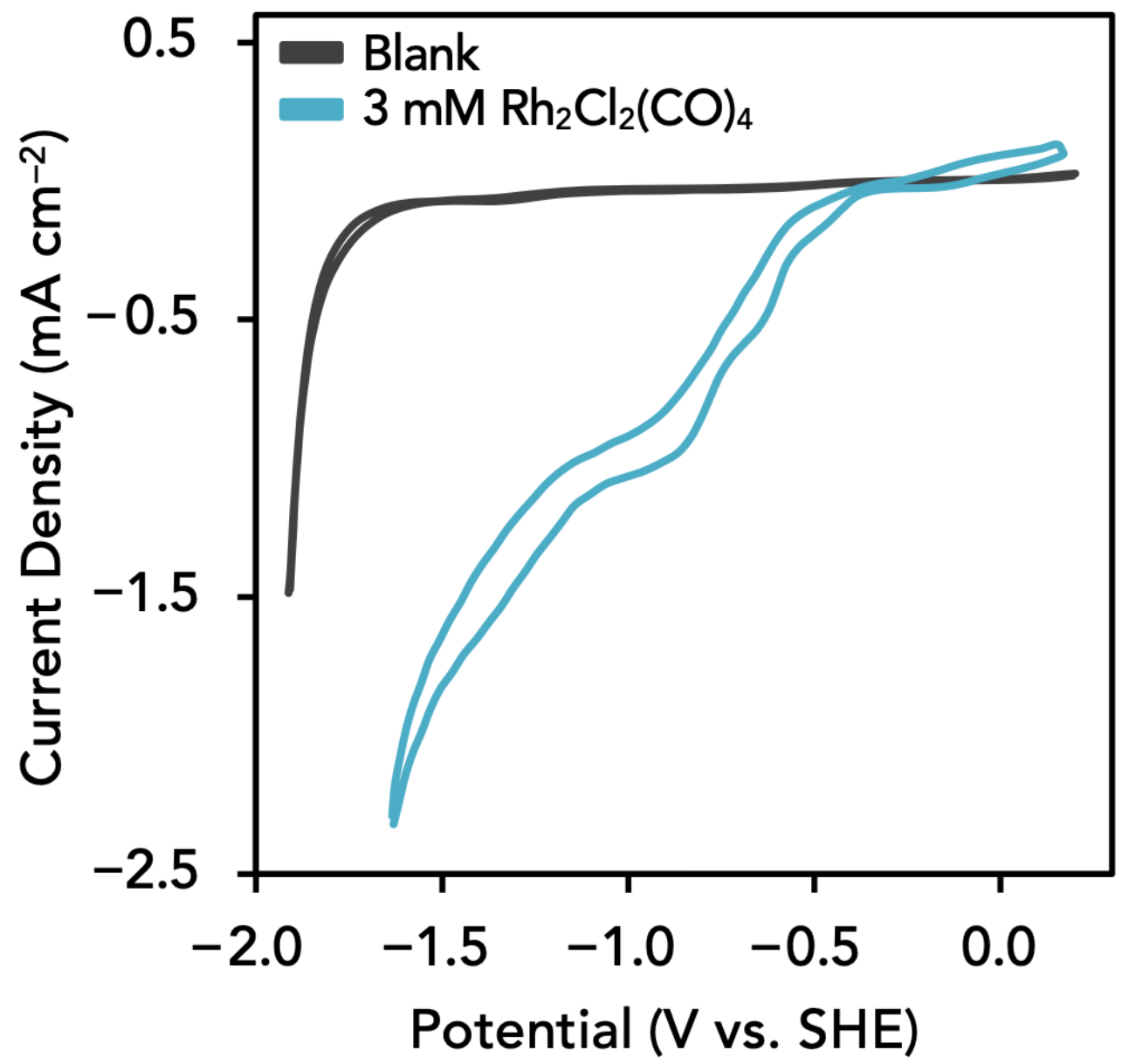

Figure S17. Cyclic voltammogram of the electrolyte with and without 3 mM $Rh_2Cl_2(CO)_4$ using a 20 mV s^{-1} scan rate. Experimental conditions: 0.52 M styrene, 0.1 M TBAOTf, 50/50 v/v IPA/H_2O, glassy carbon cathode, 5 bar CO.

Supplemental Discussion 3. Local H_2 concentration in control experiments

Bulk H_2 concentration in thermochemical control experiments
The solubility of H_2 in water and isopropanol is 0.8 and 3 mM, respectively, at 1 bar and 25 °C.[5–7] Assuming linear scaling of a gas-liquid equilibrium, the concentration of H_2 in water and isopropanol with a headspace H_2 pressure of 2.5 bar is 2 and 7.5 mM, respectively. Given that we operate with a 50/50 v/v IPA/H_2O mixture, the actual H_2 concentration likely exists between the concentrations of the pure solvents with slight deviations due to the presence of TBAOTf as the supporting electrolyte salt.

Maximum interfacial H_2 concentration in electrochemical control experiments
We can calculate the analogous interfacial H_2 concentration present in an electrochemical experiment using a transport-based calculation, where j_{lim} is the transport-limited current density, n is the number of moles of electrons, F is Faraday's constant, $D_{\mathrm{H_2}}$ is the diffusion coefficient of H_2 (5×10^{-5} $\mathrm{cm^2\ s^{-1}}$ in pure water at 25 °C)[8], $C_{\mathrm{H_2}}$ is the concentration of H_2, and δ is the boundary layer thickness[9] (**Equation S6**). The average total current density for an electrochemical experiment at 2.5 bar CO is 1.4 mA $\mathrm{cm^{-2}}$. This condition can be represented as a constant flux boundary condition applied to a mass transport boundary layer assuming a 1.4 mA $\mathrm{cm^{-2}}$ reaction flux at the cathode. If we assume all charge goes to HER, then the maximum interfacial H_2 concentration is 2.9 mM.

Equation S6. Maximum interfacial H_2 concentration calculation

$$j_{\mathrm{lim}} = \frac{nFD_{\mathrm{H_2}}C_{\mathrm{H_2}}}{\delta}$$

$$C_{\mathrm{H_2}} = \frac{\delta j_{\mathrm{lim}}}{nFD_{\mathrm{H_2}}}$$

$$C_{\mathrm{H_2}} = \frac{(2 \times 10^{-2})(\mathrm{cm}) * (1.4 \times 10^{-3})\left(\frac{\mathrm{A}}{\mathrm{cm^2}}\right) * 1000\left(\frac{\mathrm{cm^3}}{\mathrm{L}}\right)}{2(\mathrm{mol\ e^-}) * F\left(\frac{\mathrm{C}}{\mathrm{mol\ e^-}}\right) * (5 \times 10^{-5})\left(\frac{\mathrm{cm^2}}{\mathrm{s}}\right)}$$

$$C_{\mathrm{H_2}} = 2.9\ \mathrm{mM}$$

Even at similar H_2 concentrations at the cathode, the TOF during an electrochemical experiment at 2.5 bar CO is 50× larger than the TOF during a thermochemical experiment at 2.5 bar CO and 2.5 bar H_2. Taken together, these data support the conclusion that a thermochemical mechanism from electrochemically generated H_2 is not responsible for the majority of the aldehyde product detected.

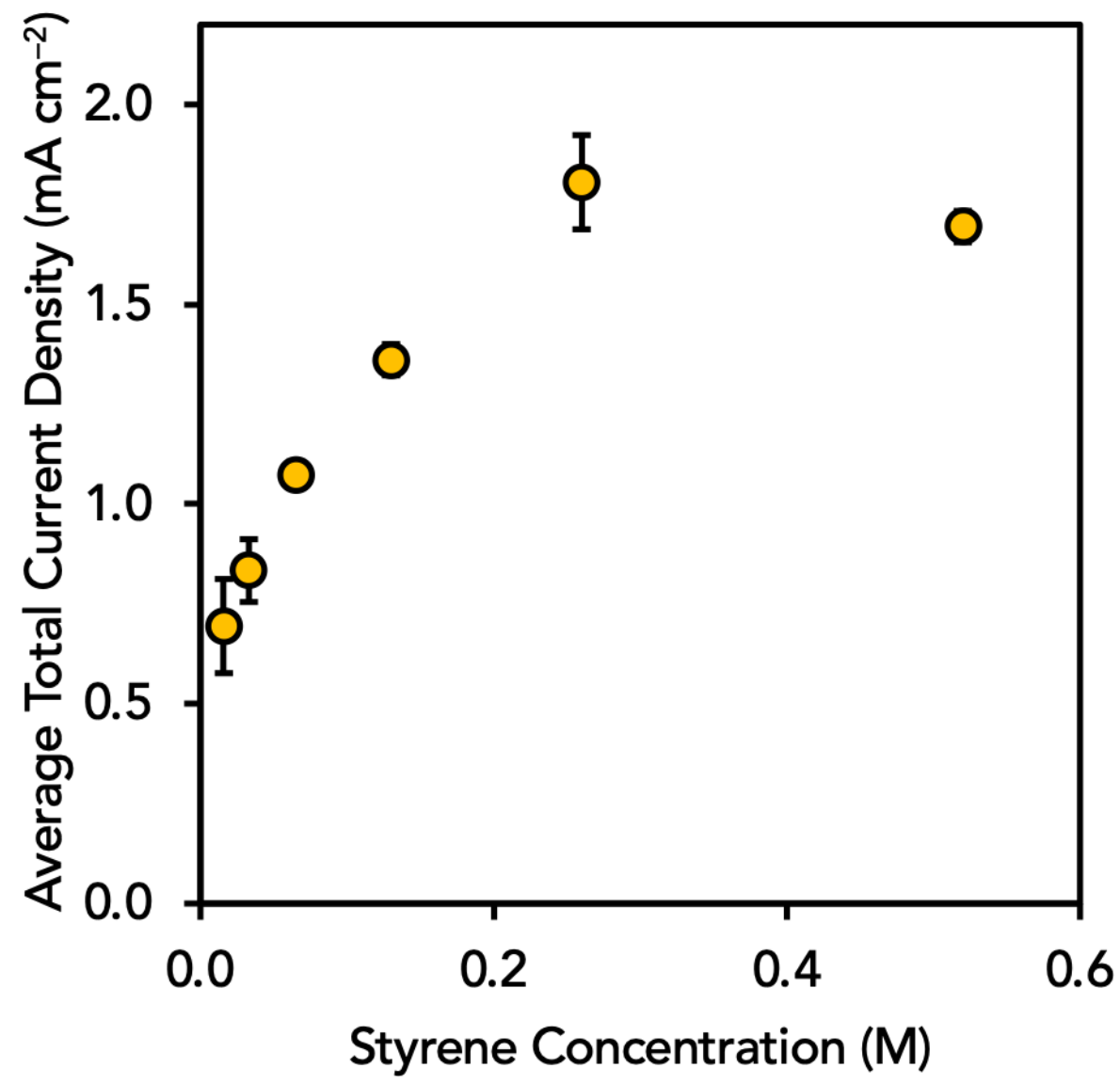

Figure S18. Average total current density as a function of styrene concentration. Error bars represent one standard deviation with n ≥ 2. Experimental conditions: 3 mM $Rh_2Cl_2(CO)_4$, combined styrene and ethylbenzene concentration of 0.52 M, 0.1 M TBAOTf, 50/50 v/v IPA/H_2O, Toray 120 (5% Teflon) carbon paper cathode, 5 bar CO, –1.11 V vs. SHE.

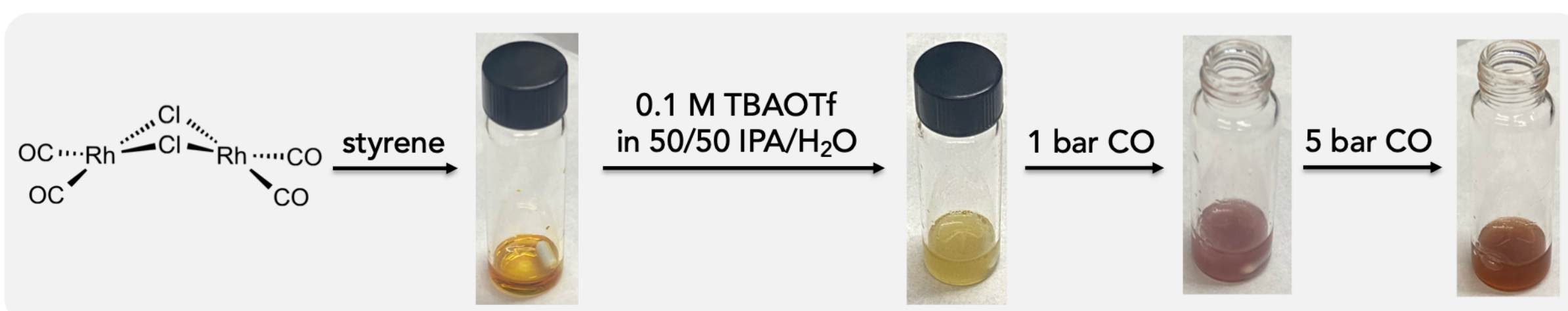

Figure S19. Color changes of $Rh_2Cl_2(CO)_4$ in the electrolyte and at 1 and 5 bar CO.

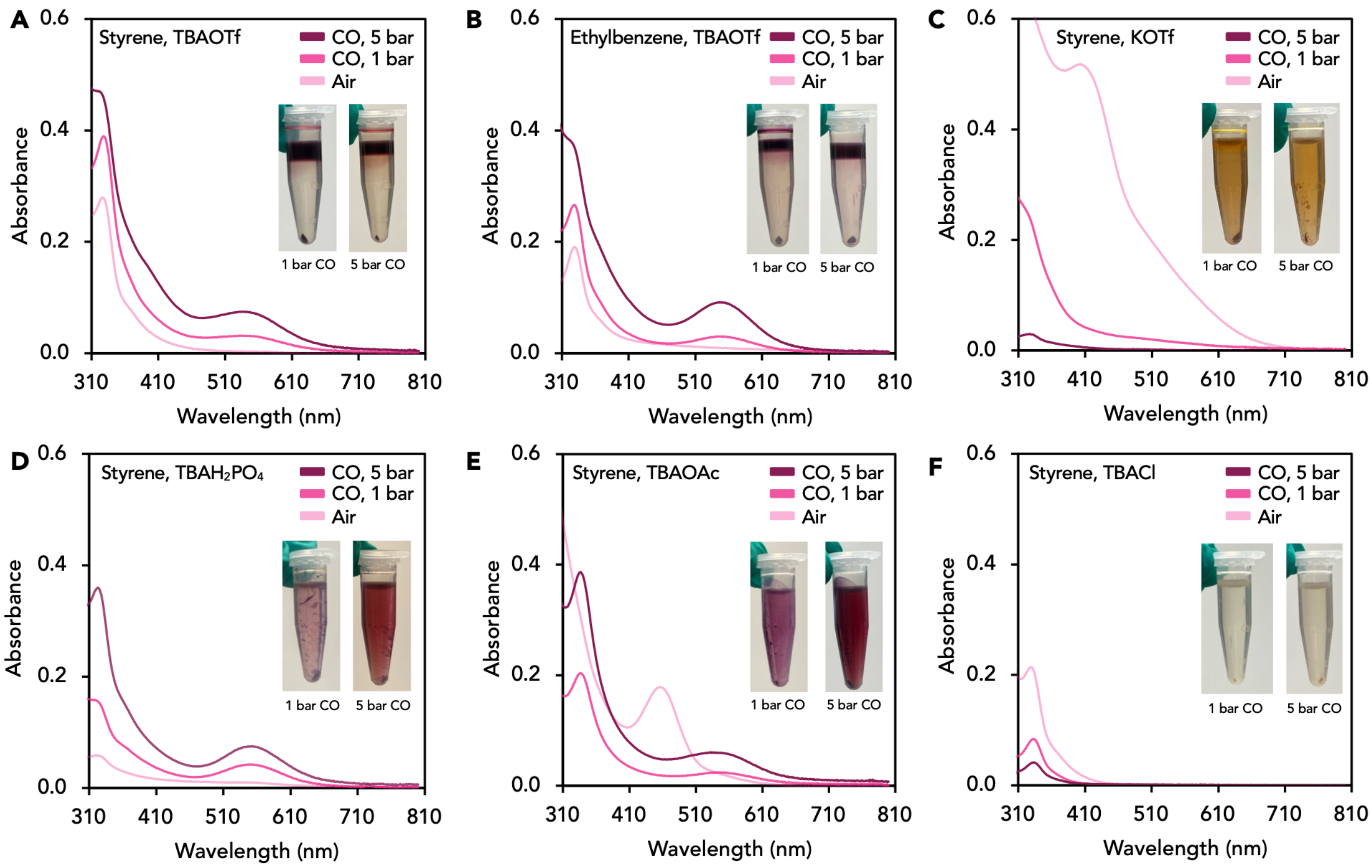

Figure S20. UV-visible spectroscopy of various electrolyte conditions in the presence of CO. **A**) Standard conditions. **B**) Ethylbenzene instead of styrene. **C**) KOTf instead of TBAOTf. **D**) $TBAH_2PO_4$ instead of TBAOTf. **E**) TBAOAc instead of TBAOTf. **F**) TBACl instead of TBAOTf. Electrolyte conditions unless otherwise stated: 3 mM $Rh_2Cl_2(CO)_4$, 0.52 M styrene, 0.1 M TBAOTf, 50/50 v/v IPA/H_2O.

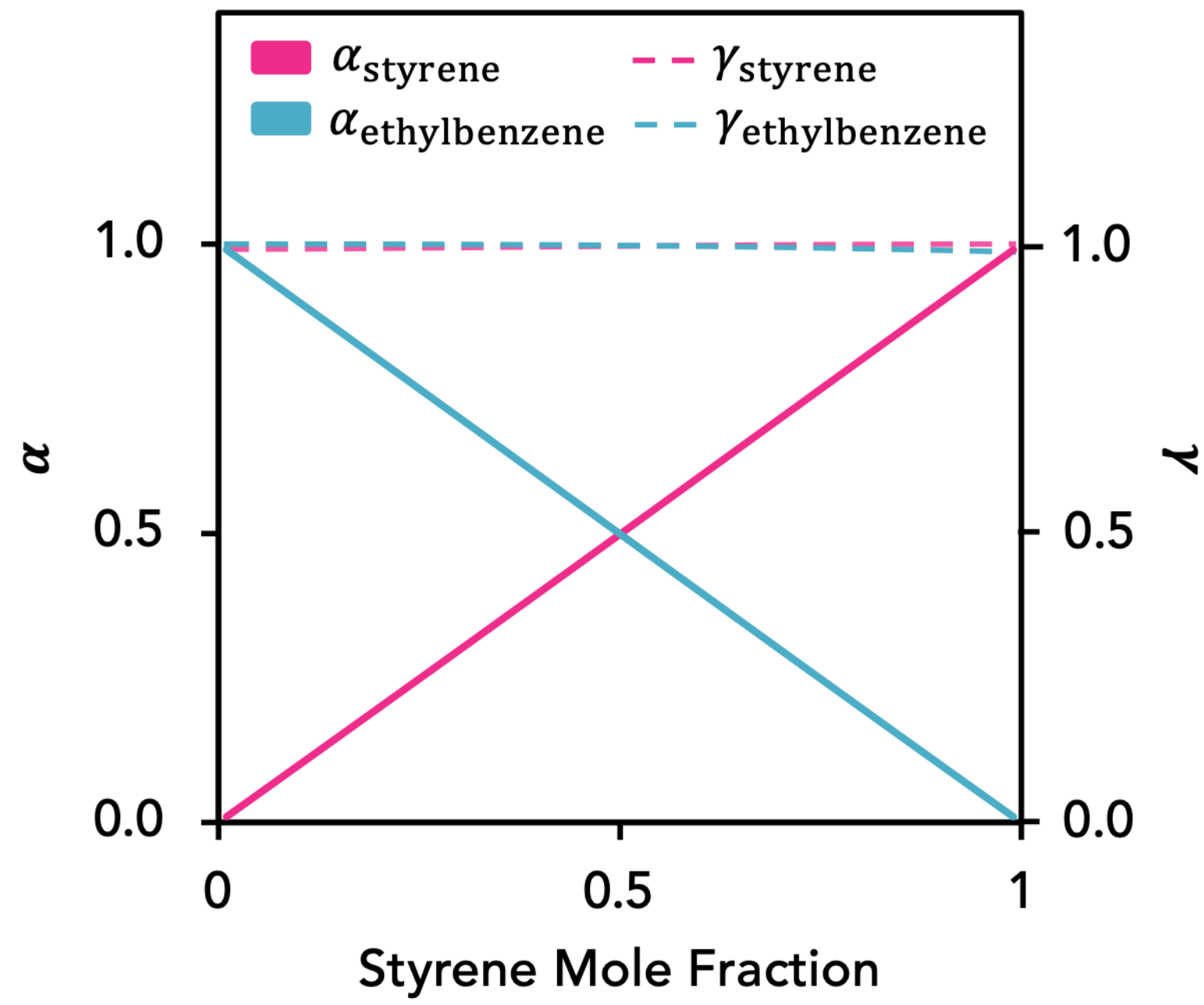

Figure S21. Activity (α) and activity coefficients (γ) of styrene and ethylbenzene in a binary mixture as predicted by a UNIFAC model.

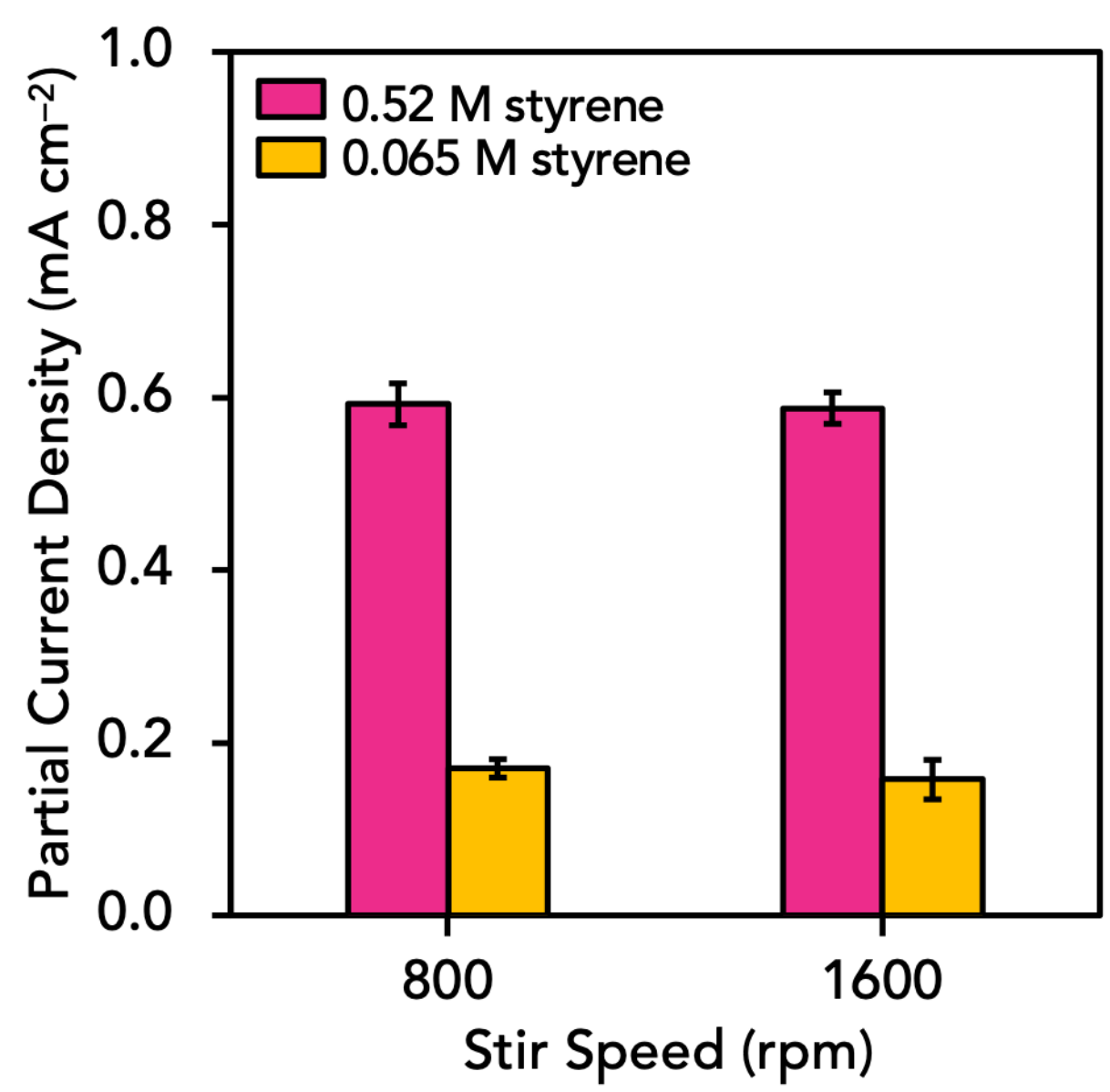

Figure S22. Partial current density stir rate dependence at 0.52 and 0.065 M styrene. Error bars represent one standard deviation with n ≥ 2. Experimental conditions: 3 mM $Rh_2Cl_2(CO)_4$, combined styrene and ethylbenzene concentration of 0.52 M, 0.1 M TBAOTf, 50/50 v/v IPA/H_2O, Toray 120 (5% Teflon) carbon paper cathode, 5 bar CO, –1.11 V vs. SHE.

Supplemental Discussion 4. Maximum transport-limited current density

The solubility of CO in water and isopropanol is 0.9 and 5 mM, respectively, at 1 bar and 25 °C.[10–12] Assuming linear scaling of a gas-liquid equilibrium, the concentration of CO in water and isopropanol with a headspace CO pressure of 5 bar is 4.5 and 25 mM, respectively. Given that we operate with a 50/50 v/v IPA/H_2O mixture, the actual CO concentration likely exists between the concentrations of the pure solvents with slight deviations due to the presence of TBAOTf as the supporting electrolyte salt. The following calculation has used the average of these solubilities.

We can calculate the maximum transport-limited current density using the following equation where j_{lim} is the transport-limited current density, n is the number of moles of electrons, F is Faraday's constant, D_{CO} is the diffusion coefficient of CO (1.8×10^{-5} $\mathrm{cm^2\ s^{-1}}$ in pure water at 25 °C),[13,14] C_{CO} is the concentration of CO, and δ is the boundary layer thickness[9] (**Equation S7**). We found that the observed plateau at 0.6 mA $\mathrm{cm^{-2}}$ is well below the CO transport limit of 2.6 mA $\mathrm{cm^{-2}}$, so the plateau is kinetic, not transport-limited.

Equation S7. Maximum transport-limited current density of CO

$$j_{\mathrm{lim}} = \frac{nFD_{\mathrm{CO}}C_{\mathrm{CO}}}{\delta}$$

$$j_{\mathrm{lim}} = \frac{2(\mathrm{mol\ e^-}) * F\left(\frac{\mathrm{C}}{\mathrm{mol\ e^-}}\right) * (1.8 \times 10^{-5})\left(\frac{\mathrm{cm^2}}{\mathrm{s}}\right) * (1.5 \times 10^{-2})\left(\frac{\mathrm{mol}}{\mathrm{L}}\right)}{(2 \times 10^{-2})(\mathrm{cm}) * (1000)\left(\frac{\mathrm{cm^3}}{\mathrm{L}}\right)}$$

$$j_{\mathrm{lim}} = 2.6\ \mathrm{mA\ cm^{-2}}$$

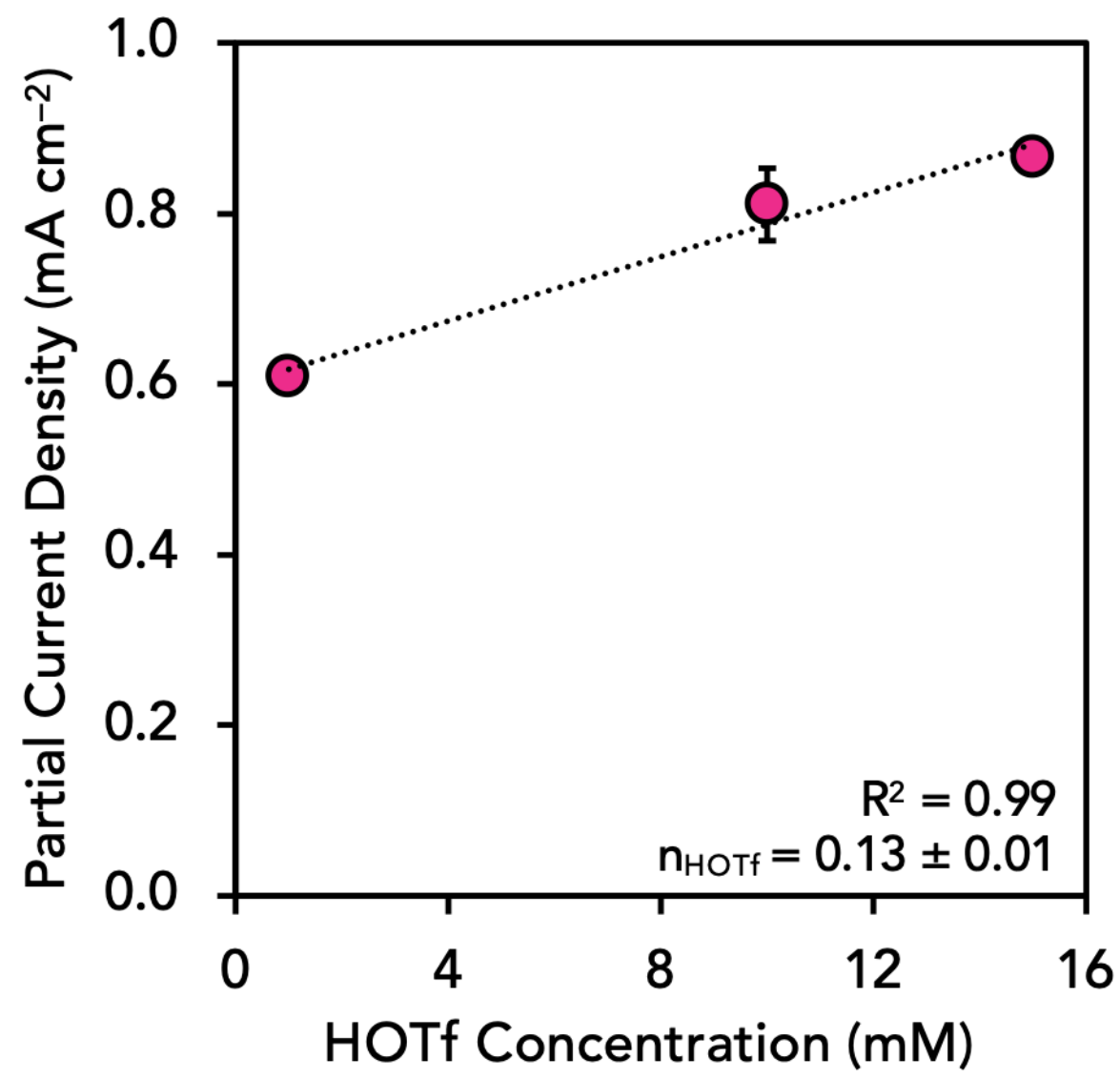

Figure S23. Partial current density HOTf concentration dependence. The uncertainty of the HOTf rate order represents the standard error of the slope of a log–log plot. Past 15 mM, significant Rh deposits on the cathode were observed. Error bars represent one standard deviation with $n \geq 2$. Experimental conditions: 3 mM $Rh_2Cl_2(CO)_4$, 0.52 M styrene, 0.1 M TBAOTf, 50/50 v/v IPA/H_2O, Toray 120 (5% Teflon) carbon paper cathode, 5 bar CO, –1.11 V vs. SHE.

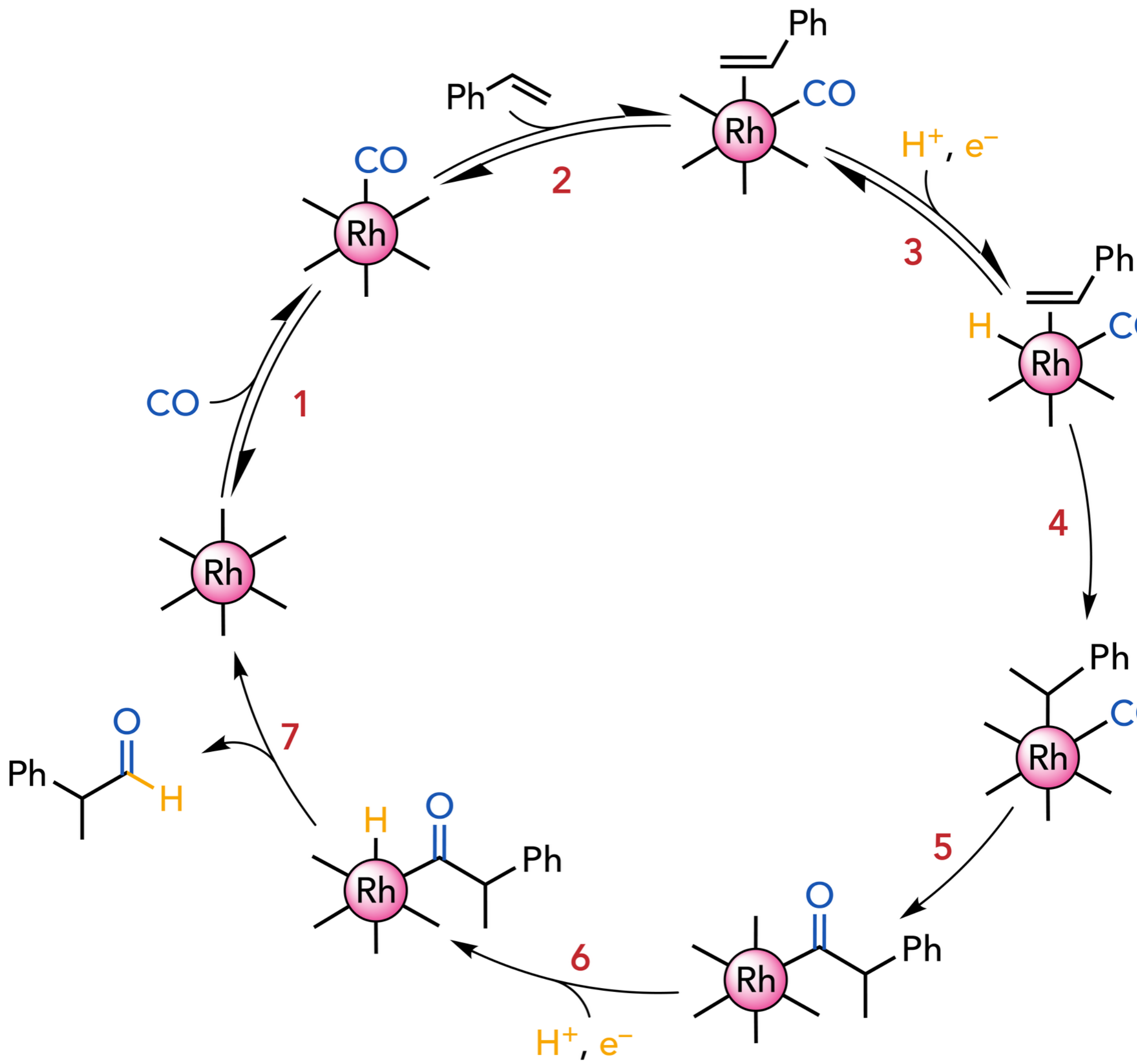

Figure S24. A possible mechanism for electro-HFN consistent with our kinetic studies. Our kinetic experiments indicate what substrates are involved before the rate determining step and which steps are rate determining in each regime. However, they do not indicate the intermediates in this cycle that follow established thermo-HFN mechanisms. **Steps 1** through **3** represent the pre-equilibrium steps identified in Figure 6, in which CO, styrene, and a hydride coordinate to the Rh center. The internal order cannot be resolved from our data. **Step 4** is the migratory insertion of the olefin into the Rh–hydride bond to form the Rh–alkyl intermediate. We assign this as a possible rate determining step at high styrene concentration and high overpotential. **Step 5** is the migratory insertion of CO into the Rh–alkyl bond to form the Rh–acyl intermediate. **Step 6** is the formation of another Rh–H from a proton and electron transfer. Finally, **Step 7** is the reductive elimination of 2-phenylpropanal and regeneration of the catalyst. Proton and electron transfers are included as proton-coupled electron transfer to form neutral intermediates. However, it is not known if these steps are concerted, and stepwise proton and electron transfers could also be consistent with our data.

References

(1) Kodintsev, I. M.; Trasatti, S.; Rubel, M.; Wieckowski, A.; Kaufher, N. X-Ray Photoelectron Spectroscopy and Electrochemical Surface Characterization of Iridium(IV) Oxide + Ruthenium(IV) Oxide Electrodes. *Langmuir* **1992**, *8* (1), 283–290. https://doi.org/10.1021/la00037a052

(2) Nishio, K.; Watanabe, Y.; Tsuchiya, T. Preparation and Properties of Electrochromic Iridium Oxide Thin Film by Sol-Gel Process. *Thin Solid Films* **1999**, *350* (1), 96–100. https://doi.org/10.1016/S0040-6090(99)00290-4

(3) Bard, A.; Faulkner, L. *Electrochemical Methods: Fundamentals and Applications*, 2nd ed.; Wiley: New York, **2001**; p 12.

(4) Yoon, Y.; Yan, B.; Surendranath, Y. Suppressing Ion Transfer Enables Versatile Measurements of Electrochemical Surface Area for Intrinsic Activity Comparisons. *J. Am. Chem. Soc.* **2018**, *140* (7), 2397–2400. https://doi.org/10.1021/jacs.7b10966

(5) Kaye, G. W. C.; Laby, T. H. *Tables of Physical and Chemical Constants*, 15th ed.; Wiley: New York, NY, **1986**.

(6) Jaatinen, S.; Touronen, J.; Karinen, R.; Uusi-Kyyny, P.; Alopaeus, V. Hydrogen Solubility in Furfural and 2-Propanol: Experiments and Modeling. *J. Chem. Thermodyn.* **2017**, *112*, 1–6. https://doi.org/10.1016/j.jct.2017.04.004

(7) Hosseini, S. Development of a Reliable Empirical Correlation to Calculate Hydrogen Solubility in Seventeen Alcoholic Media. *Sci. Rep.* **2022**, *12* (1), 9615. https://doi.org/10.1038/s41598-022-13720-1

(8) Mills, R. Self-Diffusion in Normal and Heavy Water in the Range 1-45 °C. *J. Phys. Chem.* **1973**, *77* (5), 685–688. https://doi.org/10.1021/j100624a025

(9) Williams, K.; Corbin, N.; Zeng, J.; Lazouski, N.; Yang, D.-T.; Manthiram, K. Protecting Effect of Mass Transport during Electrochemical Reduction of Oxygenated Carbon Dioxide Feedstocks. *Sustain. Energy Fuels* **2019**, *3* (5), 1225–1232. https://doi.org/10.1039/C9SE00024K

(10) Liu; Takemura, F.; Yabe, A. Solubility and Diffusivity of Carbon Monoxide in Liquid Methanol. *J. Chem. Eng. Data* **1996**, *41* (3), 589–592. https://doi.org/10.1021/je950274f

(11) Xuan, A.; Wu, Y.; Ma, P.; Sun, W.; Shen, X. Measurement and Correlation of Solubility of Carbon Monoxide in Phenol+Ethanol Solvents. *Chin. J. Chem. Eng.* **2008**, *16* (5), 762–765. https://doi.org/10.1016/S1004-9541(08)60152-4

(12) Linnemann, M.; Nikolaychuk, P. A.; Muñoz-Muñoz, Y. M.; Baumhögger, E.; Vrabec, J. Henry's Law Constant of Noble Gases in Water, Methanol, Ethanol, and Isopropanol by Experiment and Molecular Simulation. *J. Chem. Eng. Data* **2020**, *65* (3), 1180–1188. https://doi.org/10.1021/acs.jced.9b00565

(13) Poudyal, I.; Adhikari, N. P. Temperature Dependence of Diffusion Coefficient of Carbon Monoxide in Water: A Molecular Dynamics Study. *J. Mol. Liq.* **2014**, *194*, 77–84. https://doi.org/10.1016/j.molliq.2014.01.004

(14) Zhang, Y.; Geng, W.; Chen, M.; Xu, X.; Jiang, L.; Song, Y. Experimental Measurements of the Diffusion Coefficient and Effective Diffusion Coefficient of CO_2–Brine under Offshore CO_2 Storage Conditions. *Energy Fuels* **2023**, *37* (24), 19695–19703. https://doi.org/10.1021/acs.energyfuels.3c03562
